# Gray Matter NG2 Cells Display Multiple Ca^2+^-Signaling Pathways and Highly Motile Processes

**DOI:** 10.1371/journal.pone.0017575

**Published:** 2011-03-24

**Authors:** Christian Haberlandt, Amin Derouiche, Alexandra Wyczynski, Julia Haseleu, Jörg Pohle, Khalad Karram, Jacqueline Trotter, Gerald Seifert, Michael Frotscher, Christian Steinhäuser, Ronald Jabs

**Affiliations:** 1 Institute of Cellular Neurosciences, University of Bonn, Bonn, Germany; 2 Institute of Anatomy and Cell Biology, University of Freiburg, Freiburg, Germany; 3 Institute of Anatomy II, University of Frankfurt, Frankfurt am Main, Germany; 4 Department of Biomedicine, Institute of Physiology, University of Basel, Basel, Switzerland; 5 Molecular Cell Biology, Johannes Gutenberg University of Mainz, Mainz, Germany; University of California, Berkeley, United States of America

## Abstract

NG2 cells, the fourth type of glia in the mammalian CNS, receive synaptic input from neurons. The function of this innervation is unknown yet. Postsynaptic changes in intracellular Ca^2+^-concentration ([Ca^2+^]_i_) might be a possible consequence. We employed transgenic mice with fluorescently labeled NG2 cells to address this issue. To identify Ca^2+^-signaling pathways we combined patch-clamp recordings, Ca^2+^-imaging, mRNA-transcript analysis and focal pressure-application of various substances to identified NG2-cells in acute hippocampal slices. We show that activation of voltage-gated Ca^2+^-channels, Ca^2+^-permeable AMPA-receptors, and group I metabotropic glutamate-receptors provoke [Ca^2+^]_i_-elevations in NG2 cells. The Ca^2+^-influx is amplified by Ca^2+^-induced Ca^2+^-release. Minimal electrical stimulation of presynaptic neurons caused postsynaptic currents but no somatic [Ca^2+^]_i_ elevations, suggesting that [Ca^2+^]_i_ elevations in NG2 cells might be restricted to their processes. Local Ca^2+^-signaling might provoke transmitter release or changes in cell motility. To identify structural prerequisites for such a scenario, we used electron microscopy, immunostaining, mRNA-transcript analysis, and time lapse imaging. We found that NG2 cells form symmetric and asymmetric synapses with presynaptic neurons and show immunoreactivity for vesicular glutamate transporter 1. The processes are actin-based, contain ezrin but not glial filaments, microtubules or endoplasmic reticulum. Furthermore, we demonstrate that NG2 cell processes in situ are highly motile. Our findings demonstrate that gray matter NG2 cells are endowed with the cellular machinery for two-way communication with neighboring cells.

## Introduction

In addition to astrocytes, oligodendrocytes, and microglia, NG2 cells are now recognized as a fourth glial cell type in the CNS [1], [2]. NG2 cells display long narrow processes and lack gap junction coupling. Fate mapping analysis has demonstrated that in white matter the majority of NG2 cells are oligodendrocyte precursors (OPCs). In contrast, gray matter NG2 glia only rarely give rise to oligodendrocytes or astrocytes but keep their phenotype throughout postnatal life [3], but see also [4], [5].

NG2 cells are unique among glial cells in receiving synaptic input (reviewed by [2], [6]), but the physiological impact of this innervation is unknown. Specifically, it remains unclear whether pre-synaptic transmitter release generates Ca^2+^-elevations in post-synaptic NG2 cells, which might evoke cellular motility or release of neuroactive substances. This ignorance is quite astonishing in view of the increasing knowledge of glia-mediated modulation of CNS signaling, such as astrocyte-neuron interactions which gave rise to the tripartite synapse concept [7]–[9]. Moreover, it is known for more than a decade that ‘complex’ glial cells [10], which display properties similar to NG2 cells, express Ca^2+^-permeable AMPA receptors [11]–[13] and voltage-gated Ca^2+^-channels (Ca_v_s) [14]. In cultured presumed glial progenitor cells, Ca_v_s are activated by the depolarizing action of GABA [15]. However, despite these previous reports the presence of Ca_v_s in NG2 glia is still disputed. Instead, a role for the Na^+^-Ca^2+^ exchanger (NCX) in NG2 cell Ca^2+^-signaling has recently been proposed [16], [17].

There are different terms in the literature describing NG2-like cells in acute preparations of wild type or different transgenic mouse lines: complex glial cells (e.g. [10]); GluR cells (e.g. [18]), OPCs (e.g. [19]), synantocytes [20], and polydendrocytes (e.g. [21]). It is currently unknown to which degree these cellular populations overlap [6]. In the present study, we employed transgenic mice with fluorescence labeling of NG2 and GluR cells to study their process structure and Ca^2+^-signaling mechanisms. Morphological, molecular and functional analyses revealed that NG2 cells (i) generate transient elevations of the intracellular Ca^2+^-concentration ([Ca^2+^]_i_) upon different types of stimulation and (ii) display *in situ* highly motile actin-based processes.

## Results

### Cell identification and basic electrophysiological properties

Cell identification in the hippocampus was based on EYFP or EGFP fluorescence, morphology, and physiological criteria as reported previously [18], [22], [23]. Cells used for Ca^2+^-imaging (n = 836; 691 of them genotyped) were EYFP positive, had an input resistance of 193±157 MΩ, a resting membrane potential of −77±6 mV, and a membrane capacity of 33±8 pF (K^+^-based pipette solution). All cells tested (n = 23) received glutamatergic and/or GABAergic synaptic input (not shown). EYFP positive cells from homozygous (n = 351) and heterozygous (n = 340) mice did not differ with respect to the above membrane parameters, expression of Ca_v_ channel transcripts, and Ca^2+^-responsiveness upon somatic depolarization or high frequency stimulation of pre-synaptic fibers (see below for details). Therefore, data were pooled.

### Ultrastructure of neuron-NG2 cell synapses in the hippocampus

**Figure 1:**
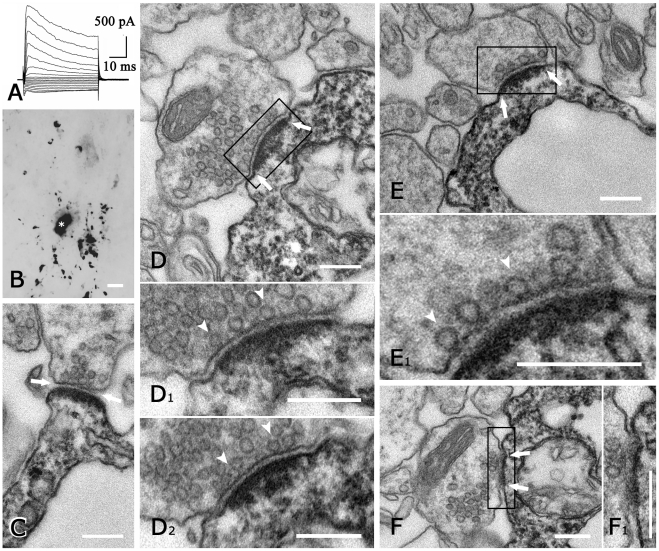
Neuron-NG2 cell synapses in mouse hippocampus. (A) Whole-cell current pattern (de- and hyperpolarization between −160 and +20 mV; 10 mV increments, holding potential −70 mV). (B) The morphology of the cell in (A) is still visible after biocytin-filling, signal conversion to DAB, and araldite-embedding for EM. Note the oval soma (asterisk) and varicose, branched processes. (C–F) Ultrastructural details of the same cell. Typical features of neuron-neuron synapses, viz. pre-synaptic vesicles, synaptic cleft (arrows) and post-synaptic density are also displayed by neuron-NG2 cell synapses, which are identified by dark DAB reaction product. Enlargements from consecutive sections of the boxed areas in (D, E, F) are shown. The synapses in (C, D_1_, D_2_, E_1_) are asymmetric, whereas that in (F_1_) is symmetric. Several pre-synaptic vesicles are docked (arrowheads in D_1_, D_2_, E_1_). Note that the diameter of post-synaptic NG2 cell processes can be very small (approx. 200 nm in C and E) or >1 µm (D). Scale bars, 5 µm (B), 200 nm (all others).

Applying correlated light and electron microscopy, we investigated synapses onto NG2 cells in the CA1 region. The typical current pattern and light microscopic morphology of the filled cells analyzed ultrastructurally (n = 3) are shown in Figs. 1A, B. Axon terminals form synapses with processes of all three NG2 cells (Fig. 1D, E). This confirms earlier findings demonstrating synapses on processes of NG2 cells in the hippocampus [6], [23]–[25]. However, only 3, 6, and 8 synapses, respectively, were found on the three cells analyzed, (Table 1), although all serial sections from a given biocytin filled NG2 cell were examined over its full process extent. The total number of synapses on the three cells was estimated to be 30 (as described above; Table 1). These synapses were very similar in structure to neuron-neuron synapses, displaying pre-synaptic vesicles, post-synaptic density and cleft material (Figs. 1C, D_1_, E_1_). In several axon terminals, docked vesicles were observed at the pre-synaptic membrane (Figs. 1D_1,2_, E_1_). In some cases, the DAB reaction product was faint enough to reveal distinct post-synaptic detail, which was indistinguishable from neuron-neuron synapses. Thus, several neuron-NG2 cell synapses could be unequivocally classified as either asymmetric (7/17) or symmetric (1/17) (see Table 1, Figs. 1D–F). All synapses were on the processes of NG2 cells, none on the soma. The post-synaptic NG2 cell process was frequently conspicuously thin, measuring 0.2–0.5 µm (Fig. 1C, E), but in several instances 1–2 µm (Fig. 1D). Thus, in contrast to earlier studies in adult rats [24], we found only few synapses per cell, and morphology in our material was indistinguishable from classical synapses between neurons.

**Table 1:** Synopsis of ultrastructural analysis of neuron-NG2 cell synapses.

cell	number of synapses observed	synaptic contacts				estimated total number of synapses
		asymm	symm	unclear	indication of perforation	
1	3	1		2		5
2	6			6	3	11
3	8	6	1	1		14
**total**	**17**	**7**	**1**	**9**		**30**

For estimation of total synapse numbers (rounded), observed numbers were multiplied by 1.75 (see text).

The physiological properties of these neuron-NG2 cell-synapses are characterized in some detail [6]. So far, however, it is largely unclear whether neuronal innervation initiates Ca^2+^-signaling in post-synaptic NG2 cells. Therefore, we tested for potential pathways provoking [Ca^2+^]_i_ elevation in NG2 cells, which might be activated by the synaptic input.

### NG2 cells express functional voltage-gated Ca^2+^-channels

**Figure 2:**
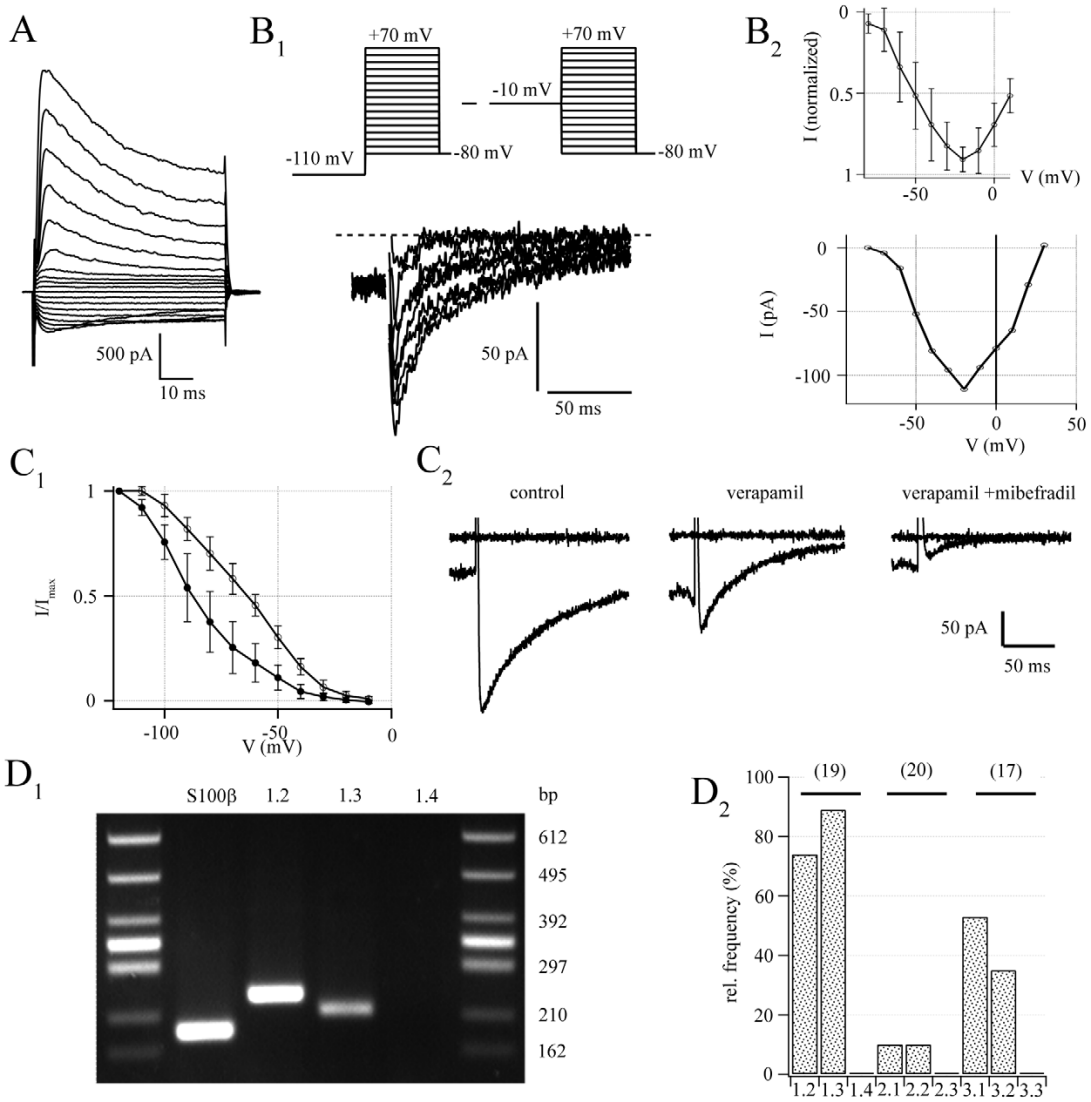
Hippocampal NG2 cells express functional Ca_v_s. (A) Typical whole-cell current pattern of an EYFP positive NG2 cell (voltage steps between −160 and +20 mV with 10 mV increment, holding potential −80 mV). This cell had an input resistance of 221 MΩ, a membrane capacitance of 23 pF, and a resting potential of −78 mV. (B) Ca_v_ currents. (B_1_) Depicted Ca_v_ currents were separated by conditioning pre-pulses (1.5 s) to −110 and −10 mV (voltage-step duration 150 ms, upper schematic) while recording in Na^+^ and K^+^ free solution containing 1 µM TTX and 10 µM SN-6. Dotted line represents zero current level. (B_2_) Current voltage relationship of 5 pooled cells (upper curve, normalized to peak) and one exemplary cell (lower curve, corresponds to B_1_) (C) Basic pharmacological properties. (C_1_) Steady state inactivation curve before (open circles) and after (filled circles) wash in of verapamil (100 µM) (C_2_) Ca^2+^ currents elicited at voltage steps to −10 mV after hyperpolarizing prepulses (−110 mV, 1.5 s, artifacts canceled for clarity). Verapamil (100 µM) reduced the initial peak current from 253 pA to 138 pA. Additional application of Mibefradil (50 µM) diminished the current to 28 pA. Upper traces represent baseline currents at −80 mV. (D) Single cell RT-PCR identified mRNA coding for different Ca_v_ subtypes. (D_1_) Representative agarose gel of mRNA-transcripts for Ca_v_ 1.2, 1.3, 1.4, and S100β. (see additional examples in Fig. S2) (D_2_) Relative abundance of Ca_v_ expression in NG2 cells. Cell numbers in parentheses.

Previous work has demonstrated that complex glial cells in wild type mice express different types of Ca_v_ [14], although later on its presence in NG2 cells has been disputed [16], [17]. To reinvestigate this issue in NG2/EYFP positive cells, putative Ca_v_ currents were isolated using Na^+^- and K^+^-free bath and pipette solutions. In addition, solutions were supplemented with Na_v_ and K_v_ channel blockers, and [Ca^2+^] in the bath was increased to 5 mM (see Materials and Methods and [14]). To remove steady-state inactivation from putative Ca_v_ channels, conditioning pre-pulses to −110 mV and −10 mV were applied for 1.5 s, respectively. Afterwards, current families were subtracted at corresponding membrane potentials. This procedure isolated transient membrane currents in NG2 cells (peak amplitudes 100±30 pA at −20 mV, n = 14) (Fig. 2B_1_). Plotting the I/V relationship of the evoked currents revealed a threshold potential of −60 mV, while peak inward currents occurred at about −20 mV (Fig. 2B_2_). The L-type channel blocker Verapamil (100 µM) reduced the maximum inward currents from 167±35 pA to 85±33 pA (n = 9, Fig. 2C_2_) and significantly shifted the half maximum voltage of the steady state inactivation curve (from −86.3±7.2 mV to −64.3±4.5 mV, n = 4, paired T-test, Fig. 2C_1_). Coapplication of the T-type channel blocker Mibefradil (50 µM) further diminished Ca_v_ currents in 4/5 cells tested (to 25±10 pA). These properties resemble Ca_v_ currents in complex glial cells of the hippocampal CA1 region [14].

To identify the subtype(s) of Ca_v_s expressed by NG2/EYFP positive cells, transcript analysis was performed employing single cell RT-PCR (Tab. S1). We found predominant expression of mRNA encoding the L-type channel isoforms Ca_v_ 1.2 and Ca_v_ 1.3 (Fig. 2D_1_) and the T-type channels Ca_v_ 3.1 and Ca_v_ 3.2. Transcripts for P/Q and N-type channels, Ca_v_ 2.1 and Ca_v_ 2.2, were less abundant, while mRNAs for Ca_v_ 1.4, Ca_v_ 2.3 and Ca_v_ 3.3 were never detected (Fig. 2D_2_). Interestingly, the majority of NG2 cells tested (n = 39/46) expressed mRNA for the glial marker S100β This is in line with our previous data showing that some of the NG2/EYFP positive cells express S100β while the astrocytic marker GFAP was consistently lacking (Karam et al., 2008).

**Figure 3:**
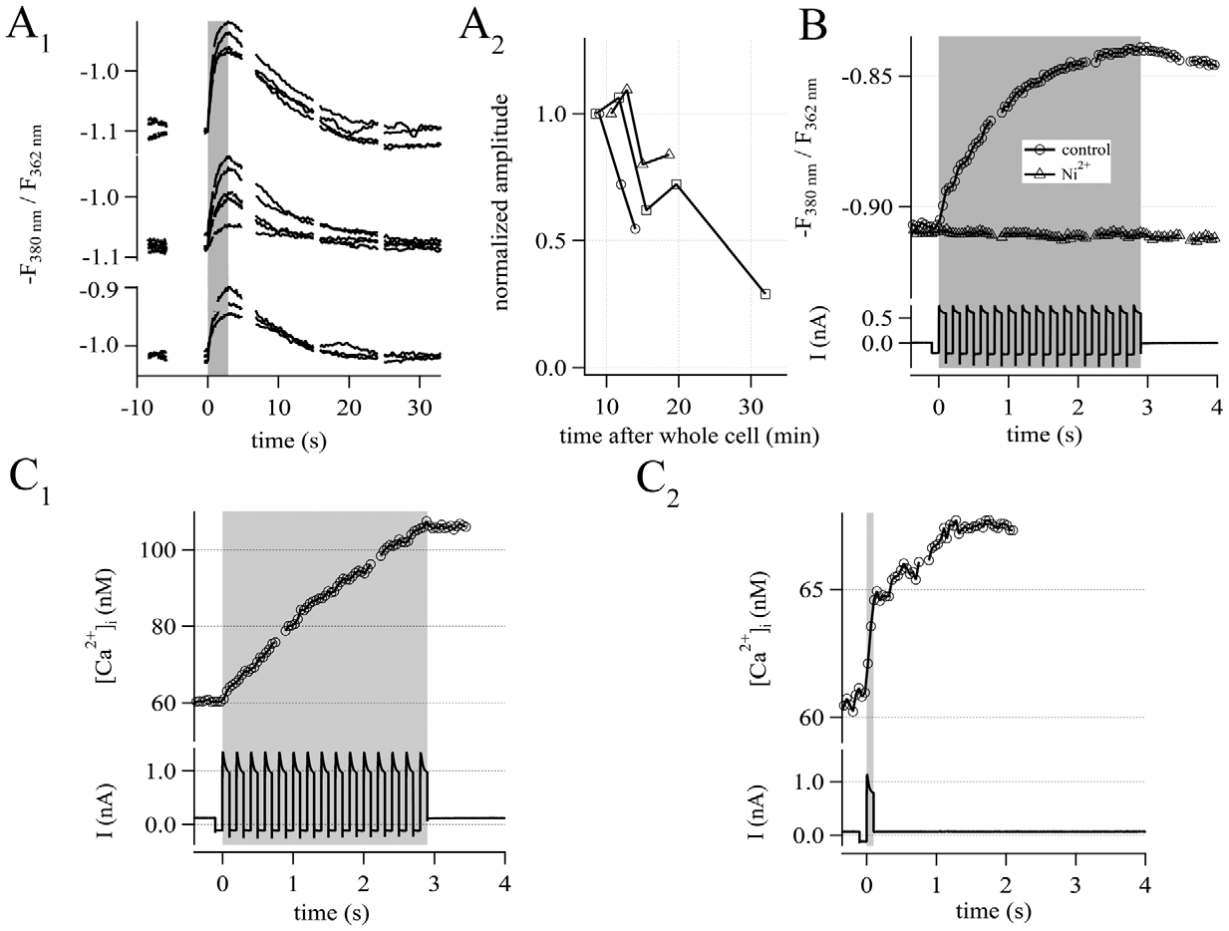
Fura-2 based calibrated Ca^2+^-imaging. (A) Depolarization of NG2 cells reproducibly generated [Ca^2+^]_i_ elevations, recorded as -F_380_/F_362_ fluorescence ratio. (A_1_) Repetitive train stimulations (15 depolarizations from −100 to +20 mV, 100 ms each; indicated by the gray box) were applied to three exemplary NG2 cells. (A_2_) Run down of the [Ca^2+^]_i_ elevation over time as revealed with successive stimulation. Amplitudes shown in (A_1_) were normalized to the first response, which was recorded 9 min after establishing the whole cell configuration. (B) 200 µM Ni^2+^ abolished the train depolarization-induced [Ca^2+^]_i_ elevations. The upper traces illustrate the [Ca^2+^]_i_ elevation in NG2 cells, before (circles) and after (triangles) application of Ni^2+^. The lower panel shows the simultaneously recorded current responses. Ca^2+^-traces represent the average of 4 cells. (C) Calibrated Ca^2+^-imaging. (C_1_) [Ca^2+^]_i_ elevations (upper traces) evoked by train stimulation (bottom). Note that the increase in [Ca^2+^]_i_ stopped immediately at the end of the stimulation (gray). Δ[Ca^2+^]_i_ amounted to 49 nM. Ca^2+^-traces represent the average of 8 cells. (C_2_) In contrast, single step depolarization (100 ms) typically elicited prolonged [Ca^2+^]_i_ elevation, outlasting depolarization (gray). Peak [Ca^2+^]_i_ was reached 1.3 s after stimulus onset and amounted to Δ[Ca^2+^]_i_(t_2_) = 8.1 nM. At the end of the depolarization the [Ca^2+^]_i_ elevation reached only 50% of the maximum (Δ[Ca^2+^]_i_(t_1_) = 4.3 nM). Ca^2+^-traces represent the average of 6 cells.

To further confirm the presence of functional Ca_v_s in NG2 cells of the hippocampus, Ca^2+^-imaging was combined with patch-clamp recording in the whole-cell mode. Train stimulation via the patch-pipette (15 consecutive depolarizing voltage steps (100 ms) from −100 mV to +20 mV, see lower traces in Fig. 3B and 3C_1_) produced reversible elevations of [Ca^2+^]_i_ in NG2/EYFP cells (Fig. 3A_1_). It is important to note that in the same cell, several [Ca^2+^]_i_ elevations could be elicited up to 30 min after establishing the whole-cell configuration (Fig. 3A_2_). Next, we tested the sensitivity of the [Ca^2+^]_i_ elevations to Ni^2+^. At high concentrations Ni^2+^ is known to non-specifically block Ca_v_s [26], [27]. Indeed, application of 200 µM Ni^2+^ abolished the [Ca^2+^]_i_ elevations in the NG2/EYFP cells tested (n = 4) (Fig. 3B).

At these high concentrations, Ni^2+^ might also inhibit the NCX [28]. To exclude that the observed block of [Ca^2+^]_i_ elevations by Ni^2+^ was due to its action on NCX rather than Ca_v_s, we tested the sensitivity of evoked [Ca^2+^]_i_ elevations to the NCX inhibitor SN-6. SN-6 has no effect on Ca_v_s while blocking NCX operating in the Ca^2+^-influx mode [29]. The amplitudes (103±34 pA vs. 86±22 pA, n = 5) and decay time-constants (39.4±6.8 ms vs. 39.2±3.6 ms, monoexponential fit, n = 4) of depolarization-induced Ca_v_ currents (at −10 mV) were not affected by SN-6 (10 µM; paired Student's T-test, p>0.05; not shown; but see Fig. 2B). Together, these data demonstrate functional expression of Ca_v_s by NG2 cells in the hippocampus, corroborating previous findings in complex glial cells of wild type mice [14].

We further analyzed the kinetics and amplitudes of depolarization-induced [Ca^2+^]_i_ elevations by Ca^2+^-imaging. Calibrated Ca^2+^-imaging measurements with Fura-2 revealed a free basal [Ca^2+^]_i_ of 60 nM. Train stimulation led to an increase in [Ca^2+^]_i_ by 49±60 nM (n = 8). The [Ca^2+^]_i_ elevation immediately ceased after the last pulse (Fig. 3C_1_). In contrast, [Ca^2+^]_i_ elevations by a single pulse considerably outlasted the pulse duration. Maximal [Ca^2+^]_i_ was observed about 1.2 s after stimulus offset. During this time Δ[Ca^2+^]_i_ almost doubled (from 4.3±1.6 nM to 8.1±1.6 nM; n = 6; Fig. 3C_2_).

**Figure 4:**
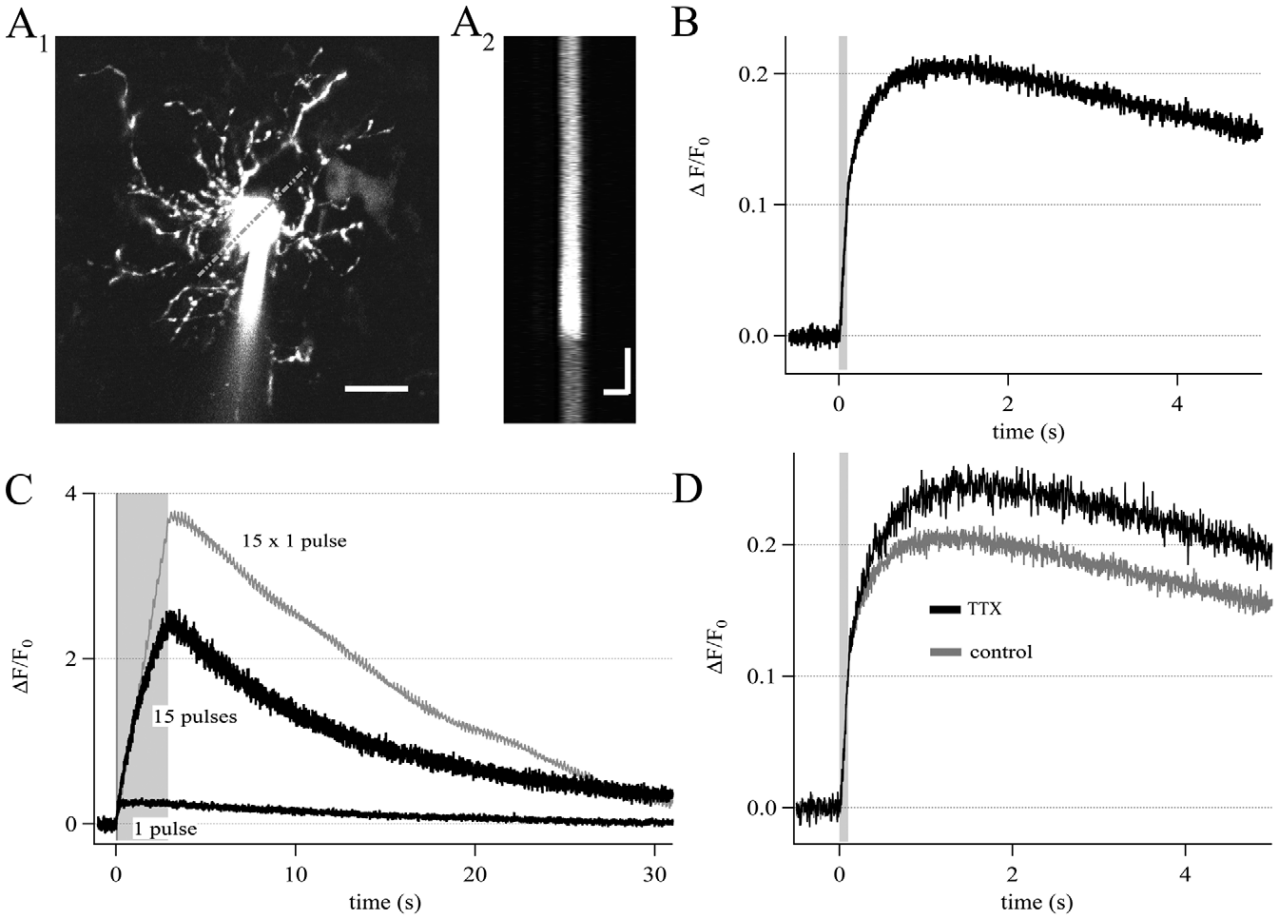
Fluo-4 based line scan Ca^2+^-imaging. (A_1_) Confocal image of a Fluo-4 loaded NG2 cell (note the tip of the patch-pipette). A single line crossing the cell soma was used for line scan imaging (gray dashed line) (scale bar 10 µm). (A_2_) Raw data of the line scan are depicted as x-t plot (scale bars 5 µm; 1 s). Fluorescence intensity of each x-line was averaged, giving one data point in the ΔF/F_0_ (t) plot. (B) Averaged Ca^2+^-traces from NG2 cells (n = 54). In each cell a single depolarization (100 ms, +20 mV, gray box) evoked somatic [Ca^2+^]_i_ elevations. [Ca^2+^]_i_ peaked 1.15 s after stimulus onset while by the end of stimulation, [Ca^2+^]_i_ reached only 50% of the maximum. (C) [Ca^2+^]_i_ elevations (black traces) evoked by single pulse (bottom) and train stimulation (gray box) together with a calculated trace (gray; response to single pulse stimulation multiplied with a factor of 15). (D) Comparison of single pulse induced Ca^2+^-responses in the presence (black, average of 13 cells) and absence (gray, average of 54 cells) of TTX (1 µM). The averaged responses did not differ significantly.

To improve time resolution of Ca^2+^-imaging we also performed LSM based x-t line scans. Therefore, individual NG2/EYFP cells were loaded with 400 µM Fluo-4 via the patch-pipette (Fig. 4A). This approach confirmed the long-lasting [Ca^2+^]_i_ elevation and its slow kinetics as observed with the calibrated Fura-2 method. During single pulses, ΔF/F_0_ increased by 0.12±0.15 (n = 70). Peak ΔF/F_0_ (0.20±0.20), however, only occurred 1.15 s after stimulus offset, and significantly exceeded the values registered at the end of the voltage step (paired Student's T-test, p<0.001). Thus, kinetics and amount of [Ca^2+^]_i_ elevation were almost the same using either imaging technique (cf. Fig. 4B, C with Fig. 3C_2_, C_1_, respectively). Obviously, there was a ceiling effect because the [Ca^2+^]_i_ elevations during train stimulation were much smaller than the calculated superposition of the responses to 15 single pulses (Fig. 4C). Saturation in [Ca^2+^]_i_ elevation and the prolonged kinetics of this signal cannot simply be ascribed to Ca^2+^ influx through Ca_v_s. First, saturation is unlikely to occur under these conditions because the limited Ca^2+^-influx during the short stimulus trains can be expected to leave the driving force for Ca^2+^ largely unchanged. Second, the [Ca^2+^]_i_ elevation outlasted channel open time more than tenfold but the binding kinetics of the Ca^2+^-indicator dyes used are in the range of microseconds [30]. Therefore, this can not account for the phenomenon.

Recently, it was suggested that in NG2 cells [Ca^2+^]_i_ elevation evoked by depolarization is mainly due to NCX operating in the Ca^2+^-influx mode in a tetrodotoxin (TTX) sensitive manner [17]. In our hands, TTX (1 µM, n = 13) neither affected the amplitudes nor the kinetics of depolarization-induced [Ca^2+^]_i_ elevations in NG2/EYFP cells (n = 13, Fig. 4D). This goes in line with our finding, that Ca_v_ channels were not influenced by the specific NCX reverse mode blocker, SN-6 (Fig. 2B).

### Ca^2+^-influx through Ca_v_s evokes Ca^2+^-induced Ca^2+^-release in NG2 cells

**Figure 5:**
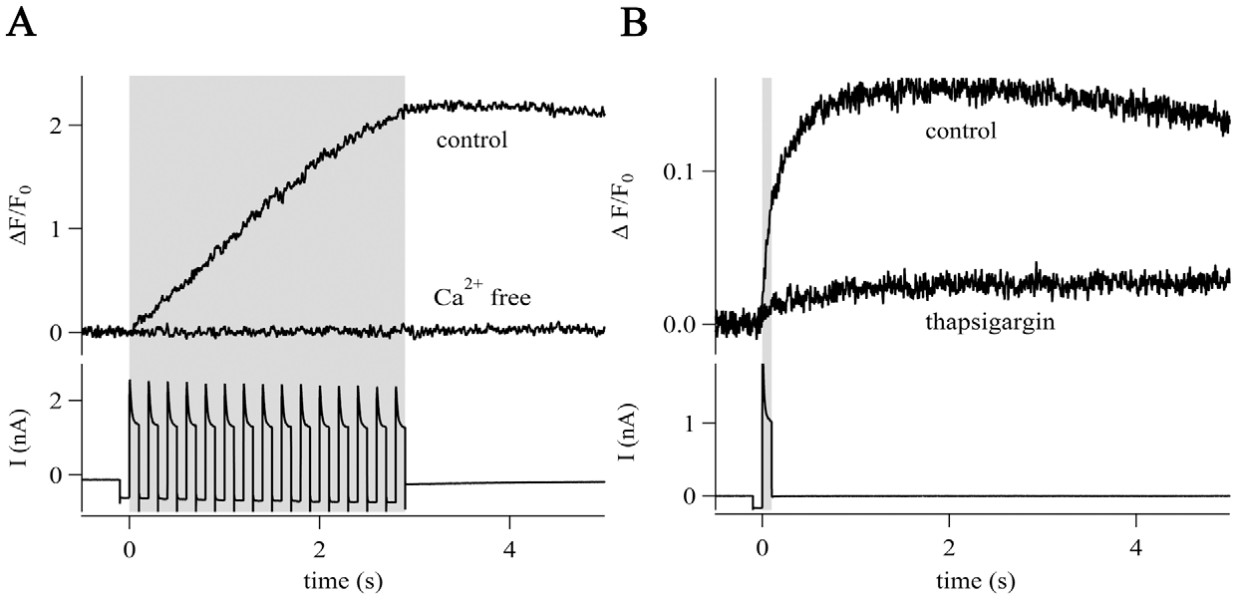
CICR in NG2 cells. (A) Train stimulation (gray box, bottom trace) evoked [Ca^2+^]_i_ elevations in the presence of Ca^2+^-containing bath solution but not in Ca^2+^-free bath solution (0 mM Ca^2+^, 2 mM EDTA). Traces represent the average of 5 cells. (B) Single pulses (gray box, lower trace) induced [Ca^2+^]_i_ elevations that were sensitive to thapsigargin (1 µM, elevation decreased to 10%) indicating a contribution of Ca^2+^-release from intracellular stores. Traces represent the average of 5 cells.

Ca^2+^-influx through the plasma membrane may evoke further increase in [Ca^2+^]_i_ by triggering Ca^2+^-release from intracellular stores [31], which might account for the observed saturation and prolonged kinetics of [Ca^2+^]_i_ elevations. To investigate whether Ca^2+^-induced Ca^2+^-release (CICR) is operative in NG2 cells we performed recordings in nominal Ca^2+^-free bath solution supplemented with 2 mM EDTA. Under these conditions no [Ca^2+^]_i_ elevation could be elicited by train stimulation. The same individual cells showed strong increases in [Ca^2+^]_i_ after switching to artificial cerebrospinal fluid (aCSF) bath solution containing 2 mM Ca^2+^ (Fura-2/CCD recording, n = 5; Fluo-4/LSM recording, n = 5) (Fig 5A). Hence, depolarization per se was insufficient to increase [Ca^2+^]_i_. This indicated that Ca_v_s mediated the initial phase of the [Ca^2+^]_i_ elevations in NG2 cells while CICR was responsible for the late phase. To test this hypothesis, single pulses were applied before and after depletion of intracellular Ca^2+^-stores. Depletion was achieved by train stimulation in the presence of thapsigargin (1 µM), a blocker of sarco/endoplasmic reticulum Ca^2+^-ATPase [32]. Under these conditions, single pulse [Ca^2+^]_i_ elevations declined to 16% of the control value (n = 5) (Fig. 5B). This suggests that the depolarization-induced [Ca^2+^]_i_ elevations in NG2 cells are due to initial influx of Ca^2+^ through Ca_v_s, followed by CICR.

### AMPA and GABA_A_ receptor-mediated depolarization evokes [Ca^2+^]_i_ elevation

**Figure 6:**
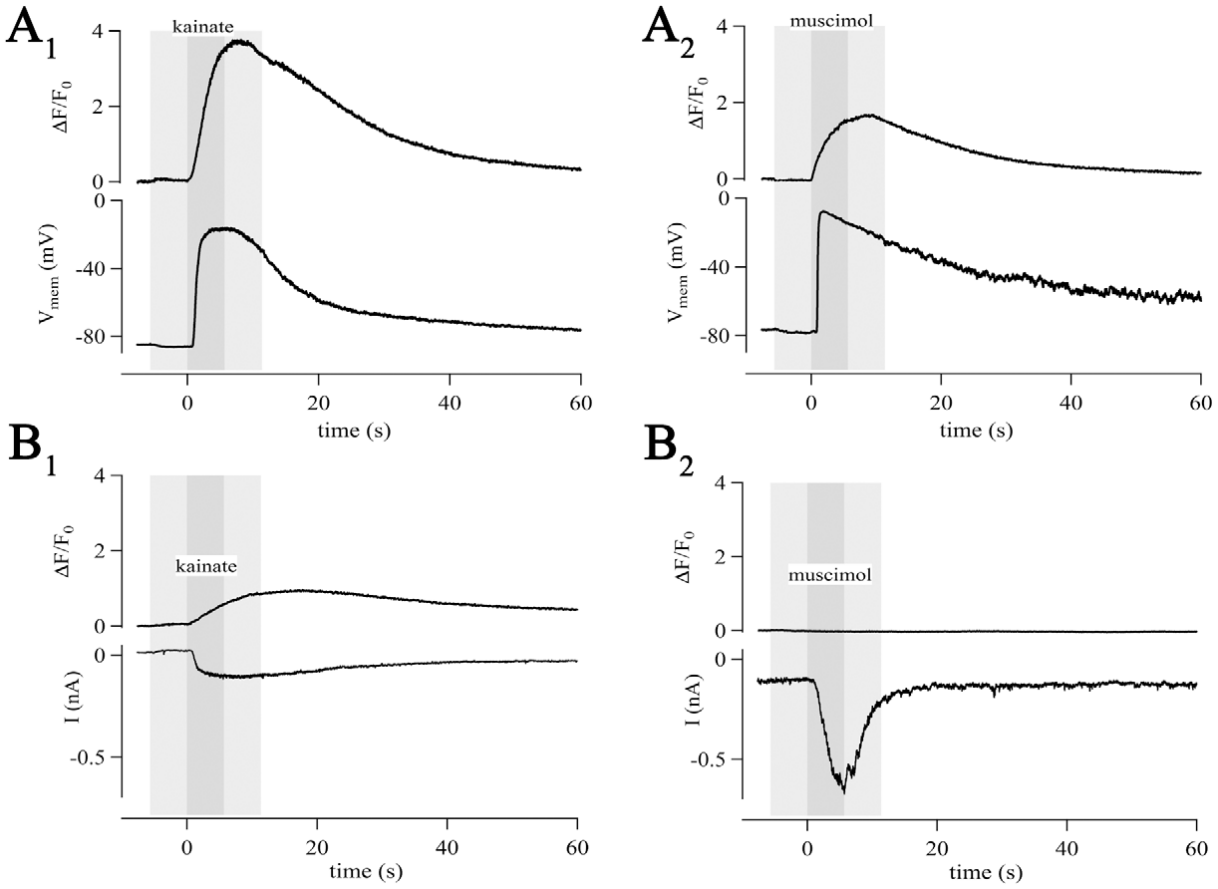
Activation of ligand-gated channels mediates Ca^2+^-responses in NG2 cells. Agonist application (dark gray boxes) was always preceded and followed by application of bath solution (light gray). (A) In the current-clamp mode, kainate (500 µM, 5.5 s, A_1_) and muscimol (250 µM, 5.5 s, A_2_) induced membrane depolarizations (lower traces; by 69 mV and 70 mV for kainate and muscimol, respectively) and [Ca^2+^]_i_ responses (ΔF/F_0_  = 3.7 and 1.7 for kainate and muscimol, respectively). (B) In the voltage-clamp mode kainate (100 µM, 5.5 s, B_1_) and muscimol (10 µM, 5.5 s, B_2_) induced inward currents (lower traces, 121 pA and 558 pA for kainate and muscimol, respectively). Ca^2+^-transients were only observed after kainate application (ΔF/F_0_  = 0.9), due to expression of Ca^2+^-permeable AMPA receptors.

Due to a relatively high [Cl^−^]_i_ in NG2 cells, activation of GABA_A_ receptors has a depolarizing effect [6]. We tested if application of AMPA or GABA_A_ receptor agonists induce elevations in [Ca^2+^]_i_ in NG2/EYFP cells. TTX (1 µM) was added to the bath solution to reduce indirect effects. In the current-clamp mode, the AMPA/kainate receptor agonist kainate (500 µM, n = 4) as well as the GABA_A_ receptor agonist muscimol (250 µM, n = 4) induced [Ca^2+^]_i_ elevations (Fig. 6A). In the voltage-clamp mode, only kainate (100 µM, n = 4) evoked increases in [Ca^2+^]_i_ (Fig. 6B_1_), due to activation of Ca^2+^-permeable AMPA receptors [11], [12], [16], [24], [33]. Muscimol (10 µM), although evoking larger inward currents than kainate, failed to affect [Ca^2+^]_i_ (n = 4) (Fig. 6B_2_). These data demonstrate that AMPA/kainate receptor activation may produce direct (Ca^2+^-influx through the receptor pore or possibly through metabotropic effects [34]) and indirect (depolarization-induced opening of Ca_v_s followed by Ca^2+^-influx) [Ca^2+^]_i_ elevations. In contrast, GABA_A_ receptor-induced Ca^2+^-influx in NG2 cells is indirect, i.e. due to membrane depolarization and Ca_v_ activation.

### NG2 cells express functional group I metabotropic glutamate receptors

**Figure 7:**
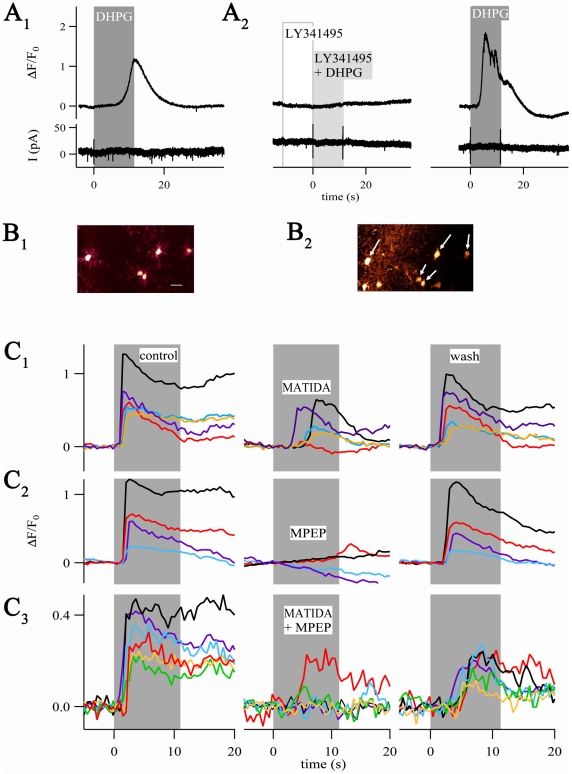
NG2 cells express mGluRs. (A_1_) The group I mGluR agonist 3,5-DHPG (100 µM, 11 s) produced [Ca^2+^]_i_ increases (upper trace) but not membrane currents (bottom). (A_2_) Pre-application of LY341495 (10 µM, 11 s) followed by co-application of 3,5-DHPG (10 µM, 11 s) completely blocked the 3,5-DHPG-induced [Ca^2+^]_i_ elevation. Three min later, 3,5-DHPG (10 µM, 11 s) again provoked a [Ca^2+^]_i_ elevation in the same cell. (B_1_) Region of interest with five NG2/EYFP cells (bar, 20 µm) which was selected for focal application of Fluo-4 AM (B_2_). Arrows mark NG2 cells from which [Ca^2+^]_i_ elevations were recorded (also seen in C_1_). Note Fluo-4 labeled, EYFP-negative cells located in the lower left corner. (C_1_–C_3_) [Ca^2+^]_i_ elevations upon 3,5-DHPG in the presence (middle) and absence (left, control; right, wash) of subtype-specific mGluR antagonists. Antagonists were pre-applied for 23 s, followed by 11 s co-application with 3,5-DHPG (10 µM, gray boxes). Applications were separated by 3 min. (C_1_) The mGluR1 specific antagonist 3-MATIDA (50 µM) mostly exerted partial block of Ca^2+^-responses. (C_2_) In most cells, 3,5-DHPG-mediated [Ca^2+^]_i_ elevations were abolished by the mGluR5 specific antagonist, MPEP (20 µM). (C_3_) In a few cells, co-application of both antagonists failed to inhibit 3,5-DHPG-induced [Ca^2+^]_i_ elevations. Experiments shown in (C_1_–C_3_) were performed in the presence of the blocking cocktail described in the text. Each row represents one individual brain slice.

Next, we tested whether NG2 cells express metabotropic glutamate receptors (mGluRs). The group I mGluR-specific agonist 3,5-DHPG was focally applied, while membrane currents and [Ca^2+^]_i_ were monitored by simultaneous patch-clamp recording in the whole cell mode and line scan imaging. All cells tested responded to 3,5-DHPG with [Ca^2+^]_i_ elevation (ΔF/F_0_ = 1.17±0.66, n = 7, 100 µM; ΔF/F_0_  = 1.14±0.79, n = 6, 10 µM). This was never accompanied by current responses (Fig. 7A_1_). The delay between substance arrival and the onset of [Ca^2+^]_i_ rises (see Material and Methods for details) varied among cells (3.4±3.3 s, n = 7, range between 0.6 and 9.4 s), but not between multiple 3,5-DHPG applications to the same individual cell.

Pre-application of the unspecific group I mGluR antagonist LY341495 [35] (10 µM, 11 s), immediately followed by co-application of 3,5-DHPG and LY341495 (11 s, 10 µM both), reversibly blocked the [Ca^2+^]_i_ elevations (n = 2, Fig. 7A_2_). Although indicating the involvement of mGluRs, these responses might have been produced indirectly, i.e. via mGluR activation of neighboring cells that innervate the NG2 cell. Another constraint of these experiments was the significant run down of the [Ca^2+^]_i_ elevations upon repetitive 3,5-DHPG applications (to 40±16% of the initial amplitudes, two applications, n = 4), probably due to wash-out of cytosolic constituents during whole cell recording. To circumvent these limitations we added TTX (1 µM) to block action potentials and inhibited P2Y receptors (with 100 µM PPADS, 100 µM suramin), mACh receptors (with 5 µM ipratropium), 5-HT2 receptors (with 10 µM methysergide), α1 receptors (with 10 µM prazosin), and GABA_B_ receptors (with 2 µM CGP55845). In addition, local loading of groups of NG2/EYFP cells with Fluo-4 AM was employed using focal pressure application (Fig. 7B_1_, B_2_). Under these conditions, almost all NG2 cells tested (96%) showed robust [Ca^2+^]_i_ elevations upon application of 3,5-DHPG (11 s; 10 µM; ΔF/F_0_ = 0.56±0.36, n = 108). Further analysis using mGluR group I subtype-specific antagonists (reviewed by [36], [37], [38]) indicated a non-uniform distribution of mGluR1 and mGluR5 in NG2/EYFP cells. The mGluR1 antagonist 3-MATIDA (50 µM) abolished the [Ca^2+^]_i_ elevations in 20% of the cells (n = 2/10; Fig. 7C_1_), while the mGluR5 antagonist MPEP (20 µM) abolished [Ca^2+^]_i_ elevations in 74% of the cells (n = 17/23; Fig. 7C_2_; for both antagonists: 23 s pre-application followed by 11 s co-application with 10 µM 3,5-DHPG). In the remaining cells, 3-MATIDA (n = 8) and MPEP (n = 6) exerted partial inhibition of 3,5-DHPG-induced responses (to 47±17%) that did not differ significantly between the antagonists. Co-application of both antagonists abolished [Ca^2+^]_i_ elevations in 88% of the NG2 cells tested (n = 15/17) (Fig. 7C_3_). The [Ca^2+^]_i_ transients recovered after wash out of the antagonists to 78±23% (n = 47) of the initial value. We noted that all cells were sensitive to at least one of the two antagonists.

### Pre-synaptic fiber tract stimulation evokes [Ca^2+^]_i_ elevations in the soma of NG2 cells

**Figure 8:**
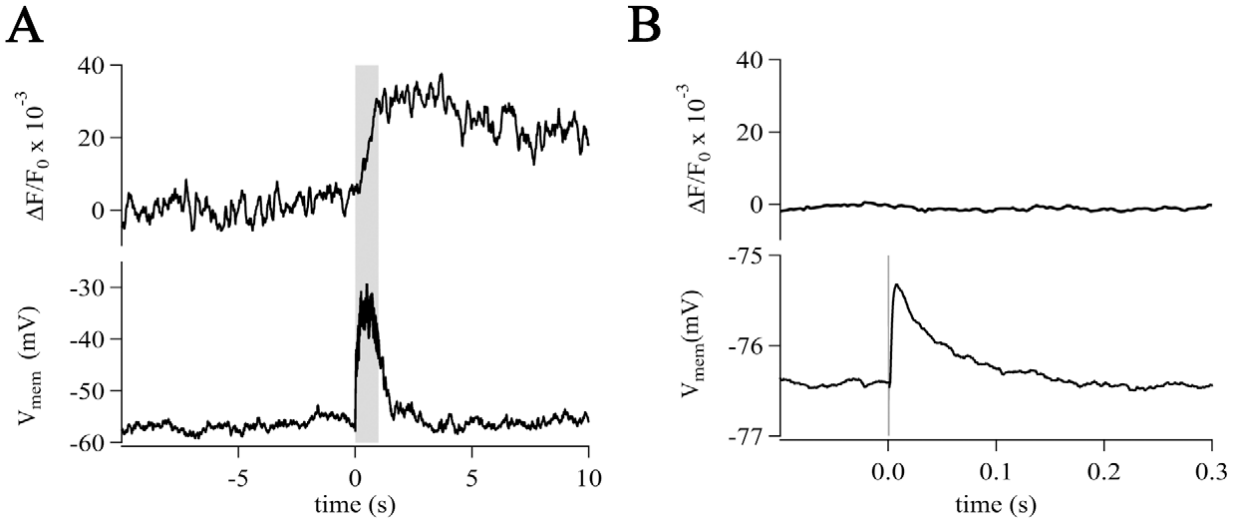
Strong excitation of pre-synaptic fiber tracts evoked small [Ca^2+^]_i_ elevations in NG2 cells. (A) Tetanic stimulation (gray box, 100 Hz for 1 s, each single pulse 200 µs, 16 V) depolarized the membrane (by 24 mV, lower trace) and provoked a small increase in [Ca^2+^]_i_ (ΔF/F_0_ = 0.04). (B) Single pulse stimulation (vertical line, 200 µs, 16 V, inter-stimulus-interval of 30 s) evoked 35 post-synaptic depolarizations (average trace depicted) and 50 failures (not shown). In this NG2 cell, the averaged depolarization (without failures) amounted to 1.1 mV (lower trace) while the corresponding [Ca^2+^]_i_ remained unchanged (upper trace, averaged).

Next, we investigated whether pre-synaptic stimulation of GABAergic interneurons or axons of glutamatergic CA3 neurons provokes [Ca^2+^]_i_ elevations in NG2 cells. Minimal stimulation induced post-synaptic currents in NG2/EYFP cells matching those observed in weakly fluorescent hGFAP/EGFP cells (previously termed GluR cells) [23] or wild type hippocampus (termed OPCs, not shown) [24], [25]. Tetanic stimulation (100 Hz, 10 s) caused robust depolarization (ΔV = 15±5 mV, n = 11) (Fig. 8A, bottom) while producing only small elevations of somatic [Ca^2+^]_i_ (ΔF/F_0_ = 0.039±0.030, n = 11). To simulate more physiological conditions, single pulses (200 µs) were applied. With this protocol, a failure rate of about 60% was observed. Excluding failures, the post-synaptic depolarization now amounted to 1.5±0.6 mV (resting membrane potential = −71±6 mV, n = 12). These depolarizations were never accompanied by somatic [Ca^2+^]_i_ elevations (n = 12) (Fig. 8B). Obviously, the sparse innervation of hippocampal NG2 cells is insufficient to provoke [Ca^2+^]_i_ elevations at the cell soma under these conditions.

### NG2 cells express vesicular glutamate transporters

**Figure 9:**
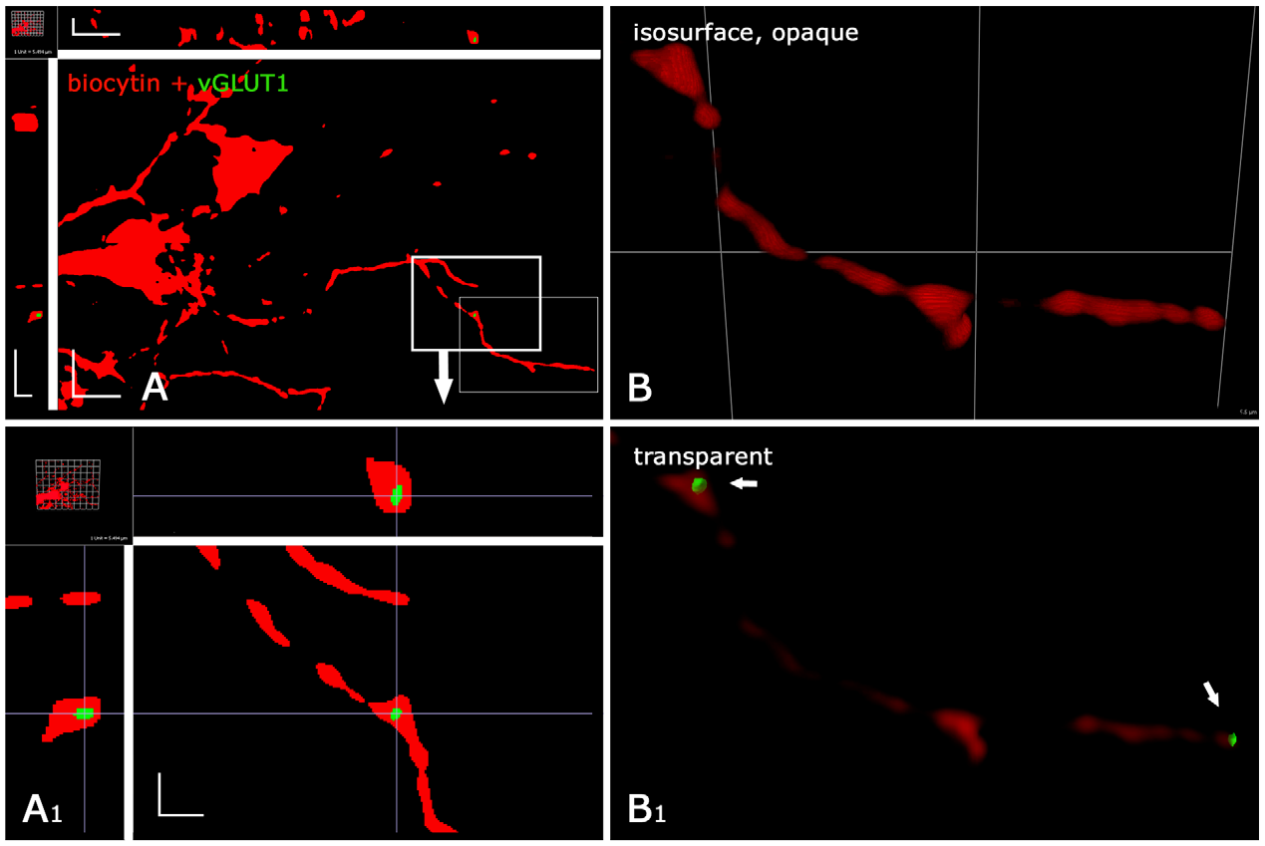
Hippocampal NG2 cells show vGLUT1-IR. NG2 cells (from hippocampal CA1, hGFAP/EGFP mouse, p 10) were identified by weak EGFP fluorescence, patch-clamp analysis, and biocytin filling visualized by CY3 (red channel), and immunoreacted for vGLUT1 (green channel). For clarity, all vGLUT-staining outside the NG2 cells was removed. (A) Analysis of a 3D stack of 75 nm optical sections after deconvolution. Several discontinuous NG2 cell processes and branching points can be seen within this section. vGLUT1-staining within one of the processes (bold line rectangle) is enlarged in (A_1_). Note that the single vGLUT1 positive object is at or below the resolution limit (approx. 200 µm, compare scale). The hairline crossings in 3D clearly indicate its localization within the NG2 cell process, which is only 0.2–1 µm wide. (B) Higher magnification of the same cell as in (A, fine line rectangle), but in 3D reconstruction and isosurface rendering. (B_1_) Several vGLUT1 positive objects (arrows) become apparent when the isosurface rendering is transparent. For a rotated, semi-transparent view of this vGLUT1-positive cell see Video S1. Scale bars (A) (x, y, z) 4.1 µm, (A_1_) 1 µm; (B) 3D grid 5.5 µm.

The observation of stimulus-induced [Ca^2+^]_i_ elevations prompted us to search for potential downstream signaling mechanisms in NG2 cells. Astrocytes express vesicular glutamate transporters (vGLUTs) in their distal processes, and were reported to communicate with neurons by Ca^2+^-dependent release of vesicular glutamate [39]–[41]. To investigate whether vGLUTs may also be expressed by NG2 cells, transcript analyses were performed. vGLUT1 and vGLUT2, but not vGLUT3 could be detected by post-recording single cell RT-PCR from NG2 cells of hGFAP/EGFP mice (p9–15). Gene transcripts for vGLUT1 were detected in 6/25 NG2 cells, resembling its prevalence in astrocytes [39]. vGLUT2 was co-expressed in 1/25 cells (not shown). As a positive control for cell type specificity, mRNA of the NG2 cell-specific PDGFα-receptor was co-amplified (n = 22). We further investigated presence and localization of vGLUT1 and vGLUT2 protein in gray matter NG2 cells in hippocampal slices by applying high resolution fluorescence microscopy, subsequent to patch-clamp recording and biocytin filling. Staining was observed for vGLUT1 (2/3 cells) and vGLUT2 (2/2 cells). Larger vGLUT1 positive puncta, putative vesicle groups, were found in the fine NG2 cell processes (Fig. 9). The inclusion of vGLUT-immunoreactivity (vGLUT-IR) within NG2 cell profiles was verified at high magnification by 3D inspection (Fig. 9A), and by increasing the opacity of surface-rendered, 3D-reconstructed NG2 cells (Fig. 9B, Video S1). Based on the rigorous thresholding, we assume that in our analysis the amount of vGLUT-IR in NG2 cells is underestimated. vGLUT1 or vGLUT2 positive puncta did not display a preference for the varicosities of NG2 cell processes but occurred all over the process tree, also at any proximo-distal distance. The immunhistochemical and RT-PCR data indicate heterogeneity among NG2 cells with regard to expression of vGLUTs.

### NG2 cell processes are motile and display actin and ezrin, but not tubulin

**Figure 10:**
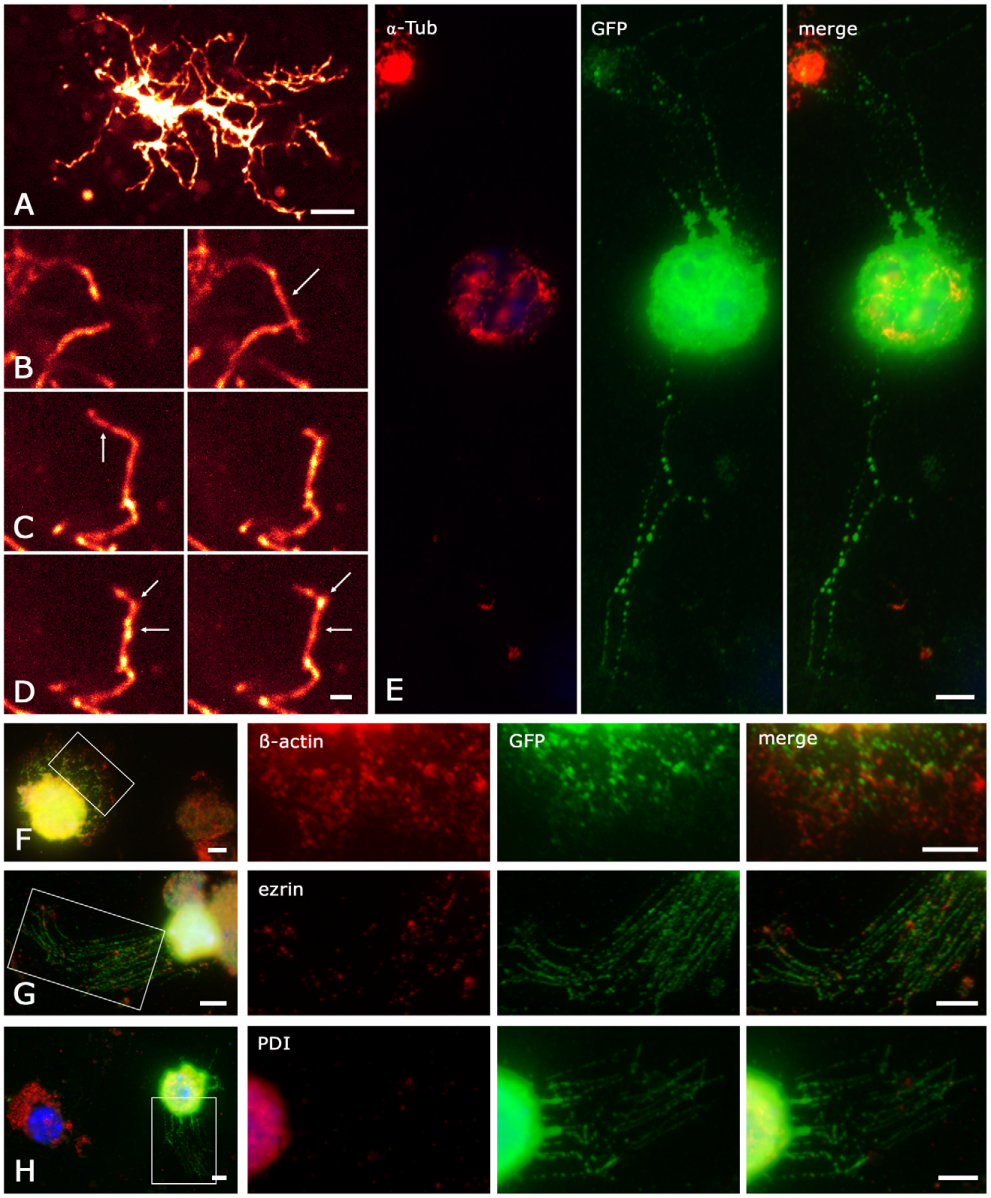
Properties of NG2 cell processes. (A–D) Two-photon time-lapse recordings. (A) Overview of an Alexa-594-labeled NG2/EYFP cell (maximum projection, 100 µm x 100 µm x 15 µm, 60 equidistant planes, scale 10 µm). (B–D) Pairs of maximum projections (16 µm x 14 µm x 5 µm, 20 planes, scale 2 µm) taken at time points t_0_ (left) and t_0_ + Δt (right). Arrows mark processes that were elongated (B, Δt = 185 s) or retracted (C, Δt = 370 s). Additionally we observed varicosities traveling along the process (D, Δt = 370 s, start and end point marked by arrows). See also Videos S2, S3. (E–H) NG2 cell processes do not contain α-tubulin and PDI. Cortical tissue from an hGFAP/EGFP mouse (p13) was freshly dissociated and quadruple-stained with a nuclear marker (bisbenzimidine, blue) and antibodies against GFAP (also blue channel), GFP (green) and one of the proteins of interest (red): α-tubulin (E), β-actin (F), ezrin (G) or the ER marker PDI (H). The cells analyzed were GFAP negative, GFP positive. Note nearby GFP negative cells (overviews, left in F–H, E). Areas boxed in the overviews (F–H) are enlarged for colocalization analysis. β-actin and ezrin were localized in the NG2 cell processes. Note the fine dimensions of these varicose processes visualized in the GFP channel (green). The PDI signal is present both in a non-identified, nearby cell (H, overview) and in the soma of the NG2 cell, but not in its processes (H, red, merge). The same is observed for α-tubulin (E red, merge). Note that α-tubulin/microtubules are well-preserved in the processes of nearby non-identified cells (E, merge) and of GFAP positive astrocytes (cf. Fig. S1). Scale bar 5 µm.

Recent reports suggested a link between [Ca^2+^]_i_ elevation and migration of NG2 cells *in vitro* [17]. To investigate the possibility of process motility *in situ*, we performed time-lapse recordings in acute hippocampal slices. We detected process motility in 5 out of 11 dye-labeled NG2/EYFP cells (Fig 10A). At least three types of process motility were observed; including elongation (Fig. 10B) and retraction (Fig. 10C) of processes (see also Videos S2, S3). Additionally, we observed that strongly dye-labeled varicosities, which are characteristic of NG2 cells, move along the processes (Fig. 10D). The varicosities traveled up to 2.9 µm within 6 min (Fig. 10D). Some varicosities showed bi-directional motility. Thus, NG2 cell processes and their varicosities exhibit motility on a minute time range.

Next, we investigated cytoskeletal constituents potentially relevant to motility of NG2 cells. Therefore, cells were freshly isolated from tg(hGFAP/EGFP) mice and selected according to their characteristic morphology and specific immunolabeling (GFP positive, GFAP negative) [18]. Antibodies against α-tubulin, β-actin, ezrin (a microvillus-associated, actin-binding protein [42]), or protein disulfide isomerase (PDI) were combined with both, anti-GFP and anti-GFAP staining. Noteworthy, α-tubulin (6/6) was not present in the processes but restricted to the soma and in a few cases to the proximal portion of processes (Fig. 10E). At the same time, the processes of nearby astrocytes were positive for α-tubulin (Fig. S1). β-actin (10/10) and ezrin (10/10) were distributed all over the cell including the fine NG2 cell processes (Fig. 10F,G). GFAP was detected in astrocytes but not in NG2 cells (36/36 cells, not shown). In the context of CICR mentioned above, we also studied the localization of endoplasmic reticulum, applying anti-PDI as a marker [43], [44]. PDI-IR (10/10) was restricted to the soma and never detected in the NG2 cell processes (Fig. 10H).

## Discussion

### NG2 cells display several mechanisms of intracellular Ca^2+^-elevation

Our data demonstrate the capability of gray matter NG2 cells to increase [Ca^2+^]_i_ via several independent pathways: G-protein coupled receptors, as well as ligand- and voltage-gated ion-channels. While the presence of mGluRs in NG2 cells represents a new finding, expression of Ca_v_s is under discussion. Recently, it was reported that NG2 cells in the hippocampus lack Ca_v_s [16], [17]. In contrast, earlier work on complex glial cells in the hippocampus described low- and high-threshold activated Ca_v_s which were sensitive to Cd^2+^ or dihydropyridines and omega-conotoxin GIVA, respectively [14]. Here, we confirm the presence of Ca_v_s in identified NG2/EYFP cells. This discrepancy with the former data may be due to different recording conditions. Ca^2+^-currents in NG2 cells are small in amplitude, compared with the dominating K^+^ currents. Its reliable separation requires use of Na^+^- and K^+^-free solutions, elevated [Ca^2+^] in the bath solution and application of conditioning pre-pulses.

The small amplitudes and high activation threshold of the Ca^2+^-currents through NG2 cell Ca_v_s raise the question of its physiological relevance. To tackle this question, we employed Ca^2+^-imaging. Using aCSF, depolarization evoked reversible [Ca^2+^]_i_ elevations in NG2 cells. This was due to influx of Ca^2+^ through Ca_v_s, but not to the activation of NCXs, as recently suggested [17]. A possible explanation for this conflicting finding might be that in the latter study, KB-R 7943 was used as an inhibitor of NCX, which blocks Ca_v_s with almost the same affinity [45]. Similarly, Ni^2+^ does not only block Ca_v_s but also NXCs [28]. SN-6, on the other hand antagonizes with high affinity only the Ca^2+^-influx mode of NCXs, preferentially of NCX1, while not interfering with Ca_v_s at the concentration used here [29]. Because (i) SN-6 did not affect the electrophysiologically recorded Ca^2+^-currents (Fig. 2B) and (ii) TTX did not diminish the voltage-step induced [Ca^2+^]_i_ elevations (Fig. 4D) we believe that in NG2 cells Ca^2+^-influx through NCXs plays only a minor role, if any. The functional characterization of the NG2 cell Ca_v_ subtypes is a challenging task for future studies. The transcript data reported here together with the pharmacological findings by Akopian [14] might provide first clues.

[Ca^2+^]_i_ elevation through Ca_v_ activation was almost doubled due to CICR. Notably, this led also to a significant prolongation of the [Ca^2+^]_i_ elevations. Thus, CICR represents a powerful mechanism to amplify small inward currents through Ca_v_s in NG2 cells. The observed saturation effect (Fig. 4C) suggests the involvement of Ca^2+^ binding sites with low affinity acting as intracellular Ca^2+^ sensors, analogously to myocardial cells (e.g. [46]). This may regulate the gain of CICR depending on ambient [Ca^2+^]_i_ levels. Currently, we do not know whether Ca^2+^ amplification exists in NG2 cell processes. The absence of PDI-IR from processes (Fig. 10H) precludes CICR in these structures, and potential amplification mechanisms would have to be independent of endoplasmic reticulum.

In agreement with previous findings [6] our data suggest the presence of Ca^2+^-permeable AMPA/kainate and GABA_A_ receptors in NG2/EYFP cells. Activation of the latter receptors depolarizes NG2 cells, which might trigger the activation of Ca_v_s. Such indirect GABA receptor-mediated [Ca^2+^]_i_ elevations have been observed in cultured OPCs [15]. Depolarizations induced by AMPA/kainate receptor activation might have similar effects, although we can not exclude a contribution of metabotropic kainate receptors to the [Ca^2+^]_i_ elevations [34]. It will be a challenge to determine whether in the fine processes, receptor activation produces depolarization sufficient for Ca_v_ activation in NG2 cells under physiological conditions.

We further report that NG2 cells in the hippocampus express functional group I mGluRs. Pharmacological analysis indicated preferential expression of mGluR5, while only a minority of the 3,5-DHPG-induced [Ca^2+^]_i_ elevations were sensitive to an mGluR1 antagonist. Whether these receptors are activated upon pre-synaptic release of glutamate needs to be demonstrated. In the present study, focusing on post-synaptic NG2 cell depolarization, fiber tract stimulation-induced [Ca^2+^]_i_ elevations have only been monitored in the soma during whole cell recording. It is very likely that dialysis of the cytosol led to an attenuation of the [Ca^2+^]_i_ elevations.

### NG2 cell processes are highly motile, actin-based surface extensions

Our live microscopic data demonstrate, for the first time, motility of NG2 cell processes *in situ*. We investigated the presence of cytoskeletal proteins in NG2 cell processes to test for prerequisites of process motility. The cytoskeleton of NG2 cell processes is found to be actin-based, since GFAP-positive glial (intermediate) filaments or microtubules were not observed by immunolabeling and electron microscopy. This appears astonishing in respect of their length (30–50 µm) and small diameter (0.2–1 µm) in between the varicose expansions. Of the many actin-binding proteins ezrin was chosen as a further marker, because its (de)phosphorylation-based mode of membrane-to-cytoskeleton linking enables rapid shape changes [47]. Ezrin, and its close relatives, radixin and moesin (the ERM protein family), are typically involved in establishing highly motile and very narrow structures in the CNS, such as neuronal growth cone filopodia [47], [48] or peripheral astrocyte processes [49], [50]. Also, ERM proteins are required for maintaining stereocilia integrity in cochlear and vestibular hair cells [51]. Altogether, the set of features displayed by NG2 cell processes classifies them as actin-based stereocilia and surface extensions. They constitute a rare example of an actin-based surface extension that is directly involved in synaptic signaling.

### Possible impact of the synaptic input onto NG2 cells

Recent findings suggest a role of neuron-NG2 cell synapses in migration. Thus, in the corpus callosum adult-born migrating NG2 cells receive glutamatergic synaptic input from demyelinated axons [52], and GABA-mediated [Ca^2+^]_i_ elevation is essential for migration of subventricular zone NG2 cells to and within white matter *in vitro* [17]. Ca_v_s might be important in this context as they have been reported to govern migration in newborn neurons, e.g. in the postnatal olfactory bulb [53]. However, the reported data relate to lesioned white matter, where neuron-glia synapses are transient [52]. In contrast, gray matter NG2 cell synapses are lesion independent and functional under physiological conditions. An alternative function of synaptic input on NG2 cells in gray matter might be the regulation of process motility uncovered here. This hypothesis would be in line with the finding that synapses were exclusively found on NG2 cell processes but not at somata.

Synaptic activation may cause small [Ca^2+^]_i_ elevations through the Ca^2+^-signaling pathways reported here. However, because the processes are devoid of endoplasmic reticulum, these [Ca^2+^]_i_ elevations are unlikely to be amplified by CICR and might occur locally confined. Local [Ca^2+^]_i_ elevations might play a role in regulation of process motility. In addition, restricted Ca^2+^-signaling might be interesting in the light of the demonstrated vGLUT expression. In neurons, vGLUT expression is sufficient for defining a glutamatergic phenotype [54]. In astrocytes vGLUTs mediate vesicular transmitter release, at least in the cell culture [39]–[41]. The scattered vGLUT organelles within NG2 cell processes might serve a similar function. The intriguing perspective that NG2 cells might signal to neighboring cells in a Ca^2+^-dependent manner remains to be addressed in future studies.

## Materials and Methods

Maintenance and handling of animals used in this study was according to local government regulations. Experiments have been approved by the State Office of North Rhine-Westphalia, Department of Nature, Environment and Consumerism (LANUV NRW, approval number 9.93.2.10.31.07.139). All measures were taken to minimize the number of animals used.

### Slice preparation

Transgenic mice with human GFAP promoter-controlled expression of EGFP (tg(hGFAP/EGFP) mice) [55] or knockin mice in which the chromophore EYFP has been inserted after the start ATG of the endogenous NG2 gene [22] aged postnatal day (p) 7–15 were anaesthetized, decapitated, and the brains were removed. Coronal hippocampal slices (200 µm thick) were cut in ice-cold oxygenated solution consisting of (mM): 87 NaCl, 2.5 KCl, 1.25 NaH_2_PO_4_, 7 MgCl_2_, 0.5 CaCl_2_, 25 NaHCO_3_, 25 glucose, 75 sucrose (347 mOsm). Slices were stored for 30 min in the same solution at 35°C and then transferred into bicarbonate-based aCSF consisting of (in mM): 126 NaCl, 3 KCl, 2 MgSO_4_, 2 CaCl_2_, 10 glucose 1.25 NaH_2_PO_4_, 26 NaHCO_3_, equilibrated with carbogen (95% O_2_ and 5% CO_2_) to a pH of 7.4 (room temperature).

### Electrophysiological recordings

Slices were transferred to a recording chamber and constantly perfused with aCSF at room temperature. Whole-cell recordings were obtained using an EPC7 or EPC8 amplifier (HEKA Elektronik, Lambrecht, Germany). The holding potential in the voltage clamp mode was −80 mV if not stated otherwise. Signals were digitized with an ITC 16 or LIH 1600 (HEKA). Patch-pipettes, fabricated from borosilicate capillaries (Hilgenberg, Malsfeld, Germany), had resistances of 4–7 MΩ when filled with a solution consisting of (in mM): 130 KCl, 2 MgCl_2_, 3 Na_2_-ATP, 5 1,2-bis(o-aminophenoxy)ethane-N,N,N',N'-tetraacetic acid (BAPTA), 10 2-(4-(2-Hydroxyethyl)- 1-piperazinyl)-ethansulfonic acid (HEPES) (pH 7.25).

For separation of Ca^2+^-currents, Na^+^- and K^+^-free bath and pipette solutions were used as described by Akopian et al. [14]. HEPES-based bath solution contained (in mM): 130 tetraethylammonium chloride (TEA), 10 HEPES, 5 CaC1_2_, 4 4-aminopyridine (4-AP), 10 glucose, supplemented with 1 µM TTX. HEPES-buffered solutions were continuously bubbled with O_2_. The pipette solution contained (in mM): 120 N-methyl-D-glucamine chloride (NMDG), 20 TEA, 0.5 CaC1_2_, 5 ethyleneglycol-bis-(β-aminoethylether) N,N'-tetraacetate (EGTA), 2 MgC1_2_, 3 Na_2_-ATP, 10 HEPES (pH 7.2). Liquid junction potentials have been corrected for.

Recordings were monitored with TIDA software (HEKA). Series and membrane resistance were checked in constant intervals with self-customized macros using Igor Pro 6 software (WaveMetrix Inc., Lake Oswedo, USA). Visual control was achieved by a microscope equipped with an infrared DIC system (Leica DM6000, Leica, Mannheim, Germany) and an IR objective (HCX APO L 20x/1.0 W; Leica). Infrared and epifluorescence images were captured with a digital CCD camera (DFC350FX R2; Leica).

Membrane currents were compensated offline for stimulus artifacts using Igor Pro 6 software according to the following procedure: Ten traces evoked by voltage steps from −80 to −70 mV were averaged and fitted monoexponentially. Compensated current traces were obtained by multiplying the fitted curve with the respective factors and subsequent subtraction from the original current traces at different membrane potentials.

Evoked post-synaptic currents in NG2 cells were compensated for stimulus artifacts by subtracting averaged failure traces.

Substances were pressure-applied focally using a multichannel Octaflow superfusion system (ALA Scientific Instruments, Farmingdale, USA). The 20–80% rise time of agonist concentration amounted to ∼100 ms. Short test pulses of GABA were used to assess the delay between valve opening and arrival of the substance at the recorded cell, which ranged between 0.4 and 0.8 s. All agonist responses were corrected for this delay. In some cases, substances were applied by changing the bath solution. All statistical data are given as mean ± SD.

### Two-photon time-lapse imaging

Individual NG2/EYFP-positive cells were filled for 2 min with Alexa-594 (Invitrogen, Karlsruhe, Germany) via the patch-pipette [56]. Dye was allowed to diffuse for >30 min before imaging. Subsequent two-photon imaging was performed on a confocal laser scanning microscope (LSM)(SP5, Leica) equipped with a mode-locked infrared laser (MaiTai BB, Newport/Spectra Physics, Irvine, USA). The dye was excited at 810 nm and emitted light was detected with built-in non-descan detectors below 680 nm. These experiments were performed at 35°C to increase process motility. The bicarbonate concentration of aCSF was reduced to 20 mM to achieve correct pH values. Image stacks of up to 60 optical planes were acquired for 20 to 60 min (z-step distance 250 nm, aCSF). We assured by inspection of all optical planes that the observed cellular motility was not caused by drift of slices, recording chamber, or microscope.

### Ca^2+^-imaging

NG2/EYFP cells in the stratum radiatum of the CA1 area were used for Ca^2+^- imaging. To determine absolute [Ca^2+^]_i_ and achieve a high time resolution of Ca^2+^-transients two different methods were applied.

(i) Changes in [Ca^2+^]_i_ were monitored by a CCD camera (SensiCam; TILL photonics, Martinsried, Germany) mounted on a wide-field epifluorescence system (Polychrome II, TILL photonics). It was attached to an upright microscope (Axioskop FS2, Zeiss, Oberkochen, Germany) equipped with a 60x LUMPlan FI/IR objective (Olympus Optical Co., Hamburg, Germany). Fluorescence excitation was achieved by a monochromator. Individual cells in acute hippocampal slices were loaded via the patch-pipette with Fura-2 (200 µM; Invitrogen). Dye filling lasted ≥5 min before Ca^2+^-imaging was started. If not stated otherwise, Fura-2 was excited at 380 or 340 nm for 40 ms and emission was detected at an acquisition rate of 25 Hz during, and 3 Hz after depolarization. Single frames were recorded at the isosbestic point (362 nm) before and after each sequence. This allowed offline calculation of pseudo-ratiometric images to correct for bleaching. The latter was assumed to be proportional to exposure time. A linear function was calculated from the first and the last 362 nm frame of each of the 380 or 340 nm sequences. This function was used to determine the 362 nm values for each recorded frame. Pseudo-ratios F_380_ or F_340_/F_362_ were calculated from the measured F_380_ or F_340_ and the extrapolated F_362_ values for each time point. F_380_/F_362_ pseudo-ratios were inversely plotted so that [Ca^2+^]_i_ elevations are always indicated by upward deflections.

Absolute [Ca^2+^]_i_ was estimated through calibration according to Grynkiewicz et al. [57]:
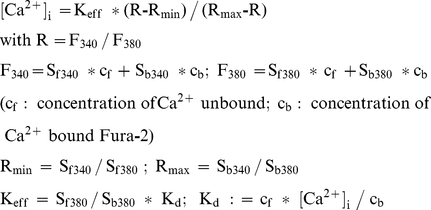



R_min_ and R_max_ were determined with 10 mM BAPTA or 10 mM CaCl_2_ in the pipette solution, respectively. K_d_ was determined with a pipette solution buffered to 11 nM free Ca^2+^ and amounted to 51 nM. R(t) curves were calculated from two successive recordings at 380 nm and 340 nm. F_380_(t) and F_340_(t) were corrected for bleaching using the pseudo-ratio method described above. Calibration was performed using self-customized IGOR 6 functions.

(ii) Alternatively, an LSM (Leica) was used for Ca^2+^-imaging, allowing for higher time resolution. Individual NG2/EYFP positive cells were loaded with Fluo-4 (400 µM, Invitrogen) via the patch-pipette. Subsequent line-scans, taken at the soma, were recorded with an excitation at 488 nm. Emission was detected between 500 and 650 nm. Signals were sampled at 1–0.4 kHz. Changes in [Ca^2+^]_i_, measured as change in fluorescence intensity (ΔF), were offline related to the baseline fluorescence (F_0_) according to ΔF/F_0_ = (F - F_0_)/F_0_. Time-correlated signals from individual cells were averaged to improve signal-to-noise ratio. For local loading of groups of EYFP positive cells, Fluo-4 AM (10 µM, Invitrogen) with 0.01% Pluronic F127 was focally pressure-applied for 5 min employing an Octaflow System (ALA Scientific Instruments). x-y-t scans of 2.2 µm thick single optical planes were recorded. ΔF/F_0_ was determined in separate regions of interest (ROIs) placed in each NG2 cell soma in the field of view. Data analysis was performed with LAS Live Data Mode (Leica) and IgorPro 6 software. 3-MATIDA, (S)-3,5-DHPG, CGP 55845, GABA, ipratropium, kainic acid, methysergide, MPEP, muscimol, PPADS, prazosin, SN-6, suramin, and thapsigargin were from Tocris (Bristol, UK)

### Fiber tract stimulation

Stimulation was performed with monopolar glass pipettes filled with aCSF. Pipette resistance ranged between 0.5 and 2 MΩ Biphasic constant voltage-pulses of 100–200 µs were applied with a stimulus generator (STG 2004, Multi-Channel-Systems, Reutlingen, Germany). High-frequency stimulation was accomplished using Mc Stimulus 2 software (Multi-Channel-Systems). Time correlation was achieved by synchronizing TTL pulses generated by the recording software (TIDA 5.22, HEKA).

### Single cell RT-PCR

After electrophysiological characterization *in situ*, the cytoplasm of individual cells was harvested under microscopic control as reported previously [18]. Reverse transcription (RT) was started after addition of RT-buffer, 10 mM DTT (final concentration; Invitrogen), 4×250 µM dNTPs (Applied Biosystems, Darmstadt, Germany), 50 µM random hexamer primer (Roche, Mannheim, Germany), 20 U RNase inhibitor (Promega, Madison, USA), and 100 U SuperscriptIII reverse transcriptase (Invitrogen). Final volume was ∼10 µl. A multiplex two-round PCR with single-cell cytosol was performed with primers for the Ca_v_ 1, Ca_v_ 2 and Ca_v_ 3 families or vesicular glutamate transporters (vGLUT) 1/2 and vGLUT3, respectively (Table S1). Primers were located in conserved regions to amplify all members of the respective family. The first PCR was performed after adding PCR buffer, MgCl_2_ (2.5 mM), and primers (200 nM each) to the reverse transcription product (final volume 50 µl). Taq polymerase (3.5 U; Invitrogen) was added after denaturation. 45 cycles were performed (denaturation at 94°C, 25 s; annealing at 49°C, first five cycles: 2 min, remaining cycles: 45 s; extension at 72°C, 25 s; final elongation at 72°C, 7 min). An aliquot (2 µl) of the PCR product was used as a template for the second PCR (35 cycles; annealing at 54°C, first five cycles: 2 min, remaining cycles: 45 s) using nested, subunit-specific primers (Table S1). The conditions were the same as described for the first PCR-round, but dNTPs (4×50 µM) and Platinum Taq polymerase (2.5 U; Invitrogen) were added. Products were identified by gel electrophoresis using a molecular weight marker (Phi X174 HincII digest; Eurogentec, Seraing, Belgium).

Primer specificity was tested with total RNA from freshly isolated mouse brain (p20). For optimization, a two-round RT-PCR was performed with 2 ng of total RNA and primers as described above. Subsequent gel analysis did not detect unspecific products. The primers for different targets were located on different exons to prevent amplification of genomic DNA. Omission of the RT-enzyme and substitution of template by bath solution served as negative controls for reverse transcription and PCR amplification and confirmed the specificity of the reaction.

### Electron microscopy

Acute hippocampal slices were prepared from juvenile (p9–12) hGFAP-EGFP mice. Weakly fluorescent cells with a typical electrophysiological current-pattern (previously termed GluR cells; [18]) were filled with biocytin (0.5%) via the patch-pipette during whole-cell recording. Slices were then fixed for 2 h in a solution containing paraformaldehyde (PFA) and glutaraldehyde (2% each in 0.1 M phosphate buffer, PB). Fixation delay after decapitation ranged from 45–120 min. Slices containing a biocytin-filled cell were rinsed, cryoprotected in sucrose solution (30% in PB), snap-frozen in liquid nitrogen and thawed [58]. Cells were visualized for correlating light and electron microscopy by overnight incubation in a combination of avidin-biotin complex (1∶100, Vector, Burlingame, USA; [59]) and streptavidin-CY3 (1∶1,000, Vector). After rinsing, the biocytin-filled cells were coverslipped in PB and documented by recording image z-stacks under a fluorescence microscope. Subsequently, the peroxidase was developed by diaminobenzidine (DAB) and 0.07% H_2_O_2,_ for ultrastructural staining. Sections were osmicated (1% OsO4), block stained (1% uranyl acetate in 70% ethanol), dehydrated and flat embedded in Araldite. Ultrathin sections were contrasted with lead citrate and uranyl acetate. To analyze overall synaptic contacts on NG2 cells at the ultrastructural level, these flat embedded cells were completely sectioned. Inspecting all ultrathin sections from a given cell, the complete process tree was scanned for synapses on DAB-containing profiles. Most synapses found in one section could also be documented in subsequent sections. To estimate the total number of synapses, the observed number of synapses was documented (Table 1) and then multiplied by 1.75 (1+0.5+0.25). An estimated factor of 0.5 was introduced to account for the missed, nearly tangentially sectioned synapses above and below a DAB-labeled profile. This corresponds to missing unrecognized synaptic profiles which are obliquely sectioned between 30 and 0 degrees (tangential). Further, we amply estimated to have overlooked ¼ of the NG2 cell profiles, because most synapse-bearing profiles were below 0.3 µm (comp. Figs. 1 C, E), which was corrected for by a factor 0.25.

### Dissociation of NG2 cells

Unequivocal determination of antigen presence in the NG2 cell processes is hampered by light microscopic resolution because they are frequently only 200–500 nm thick. We either studied freshly dissociated NG2 cells by conventional immunofluorescence or NG2 cells in brain slices using deconvolution microscopy with higher resolution.

The isolation method applied adapts previous cell-isolation protocols [60]–[63] to permit dissociation of glial cells within 2–3 h with morphological preservation of their thin processes. Briefly, hGFAP/EGFP mice at p13–15 were anaesthetized using isoflurane and decapitated. Cortical vibratome sections were incubated for 10 min at 37°C in papain solution (20 units/ml papain, 1 mM L-cysteine, 0.5 mM ethylenediaminetetraacetate (EDTA) in Ca^2+^/Mg^2+^-containing EBSS, Worthington Biochemical Corporation, Lakewood, USA). Subsequently, sections were disaggregated using pipettes, centrifuged, and resuspended in inhibitor solution (1 mg/ml ovomucoid, 1 mg/ml BSA, 0.0005% DNase I in Ca^2+^/Mg^2+^-containing EBSS, Worthington Biochemical Corporation). Finally, the cells were centrifuged onto silane-coated slides and immediately fixed with 4% PFA.

### Immunofluorescence and microscopy

Dissociated cells on slides were quadruple-stained; incubation was with a mixture of the three primary or secondary antibodies according to standard procedures. The primary antibodies were chicken anti-GFAP (1∶500, Chemicon/Millipore, Billerica, USA), sheep anti-GFP (1∶4,000, Serotec, Düsseldorf, Germany), and a label for the protein of interest, viz. mouse anti-ezrin (1∶500, Sigma, Deisenhofen, Germany), mouse anti-α-tubulin (1∶500, Sigma), mouse anti-β-actin (1∶500, Sigma), or rabbit anti-PDI (1∶200; Stressgen, Assay Designs, Ann Arbour, USA). For cell identification and nucleus localization, AMCA-coupled donkey anti-chicken (1∶100) and dylight488-coupled donkey anti-sheep (1∶100) were combined with bisbenzimidine (1∶200,000). For visualization of the antigen of interest, cells were incubated with CY3 coupled to donkey anti-rabbit (1∶250) or anti-mouse (1∶250). NG2 cell identification was based on morphology (small soma, multiple, very thin processes directly emanating from the soma), presence of staining with anti-GFP but absence of staining with anti-GFAP [18]. GFAP-positive nearby astrocytes served as a positive control. These specimens were documented using a fluorescence microscope (Axiophot, Zeiss), controlled by Metaview software (Molecular Devices, Sunnyvale, USA) and equipped with 100×1.3, and 40×0.75 (Plan-Neofluar) lenses, and a 4 MP b/w camera (Spot Insight, KAI4021M; Diagnostic Instruments, Sterling Heights, USA).

To detect putative glutamate vesicles in NG2 cells *in situ*, vGLUT1 or vGLUT2 immunofluorescence was combined with fluorescence detection of biotycin-filled NG2 cells. Cells were identified and filled as above, and fixed in 4% PFA (in PB, 2 h). After freeze-thawing, the sections were incubated with streptavidin-CY3 (1∶1,000, overnight). Subsequent immunostaining was carried out by incubating sequentially with normal goat serum (10% in PB including 0.2% Triton X100, 30 min), rabbit anti-vGLUT2 (1∶2,000 including 0.2% TritonX100, overnight, Synaptic Systems, Göttingen, Germany), and goat anti-rabbit-Alexa 647 (1∶100, Invitrogen). For visualization of vGLUT1, only rabbit anti-vGLUT1 directly coupled to Oyster-645 (1∶200, Synaptic Systems) was applied overnight.

Detection of vGLUT-IR in NG2 cells is challenging because it is abundant and dense in brain, and NG2 cell processes are frequently thinner than 0.5 µm, as observed in the electron microscope (cf. Fig. 1C). We carried out subresolution microscopy on an appropriate microscopy setup (Zeiss 200M; Orca AG camera, Hamamatsu, Hamamatsu City, Shizuoka, Japan; Openlab software, Improvision, Coventry, UK; 40×1.3, 63×1.4, 100×1.45 oil immersion lenses, Zeiss). We applied on-chip magnification (100–160x), imaging the cells at 50–100 nm steps in two fluorescence channels (filter sets (I) ex 475/20, bp 495, em 513/17 and (II) 632/22, 660, 700/75). The resulting image stacks underwent iterative deconvolution (Openlabs) based on calculated point spread function that has previously been applied and validated for antigen colocalization in single vesicles [40], [64]. Image analysis and 3D reconstruction (Openlabs) included intensity thresholding in both channels. In particular, intensity thresholding in the vGLUT channel was rigorous and led to disappearance of most smaller vGLUT-positive puncta, with many false negatives to avoid false positives. Thresholding in the GFP channel frequently resulted in discontinuous glial cell processes. Post hoc exclusion of all vGLUT-IR outside the cell facilitated visualization. All instances of vGLUT-IR within in the glial cells were checked for full inclusion in 3D cardbox view (see Fig. 9). No vGLUT-IR was detected in controls without primary antibody. Further processing of electron or light microscopic images was done with Photoshop (Adobe Systems), and comprised only linear operations for optimizing brightness and contrast, but no selective processing of image detail.

## Supporting Information

Figure S1 **Microtubules are well-preserved in the processes of freshly dissociated, identified astrocytes.** Labeling for both, cell nuclei (bisbenzimidine) and glial filaments (GFAP, Alexa 360) is revealed in the blue channel. An astrocyte (center) and two unidentified cells (right) are displayed. Microtubules (α-tubulin, red) are obvious in the astrocyte processes demonstrating that the dissociation method does not interfere with microtubule integrity even in the processes.
(TIF)

Figure S2 **Exemplary agarose gels of mRNA-transcripts for Ca_v_ channel family and S100β.**
(TIF)

Table S1 **Primers used for single-cell RT-PCR.**
(DOC)

Video S1 **Demonstration of full inclusion of vGLUT1 positive objects in NG2 cell processes (3D reconstruction).** The cell is the one shown in Fig. 9. NG2 cells from hippocampus (CA1) were identified by electrophysiology, biocytin-filled, fixed and visualized by streptavidin CY3 (red channel). The green channel displays immunocytochemical detection of vGLUT1. For clarity, all vGLUT staining outside the cells has been removed. After deconvolution of 75 nm optical sections, the cells (n = 5) were 3D reconstructed and isosurface-rendered. Due to high magnification, a frame displays only parts of a cell. By 3D rotating the reconstruction and changing its transparency, the movies demonstrate full inclusion of the vGLUT1 objects in the small processes (<0.5 µm, often 0.2 µm). Unit of the 3D grid scale: 5.5 µm.
(AVI)

Video S2 **Elongation of an NG2 cell process.** (cf. Fig. 10B). Two-photon time-lapse video was obtained from Alexa-594 dye-loaded NG2/EYFP cell processes located in an acute brain slice. Optical stacks of 20 planes were recorded every 34 s. Maximum z-projections are shown with 1 frame per second (volume 16×14×5 µm, total time 330 s, aCSF, 35°C).
(AVI)

Video S3 **Retraction of an NG2 cell process and movement of intracellular varicosities.** (cf. Fig. 10C, D). Similar recording parameters as in Video S2 were used.
(AVI)
