# Supplementary figures and images for: Gray Matter NG2 Cells Display Multiple Ca2+-Signaling Pathways and Highly Motile Processes
Source: PLoS One. 2011 Mar 24;6(3):e17575. doi: 10.1371/journal.pone.0017575 (PMC3063786; doi:10.1371/journal.pone.0017575)

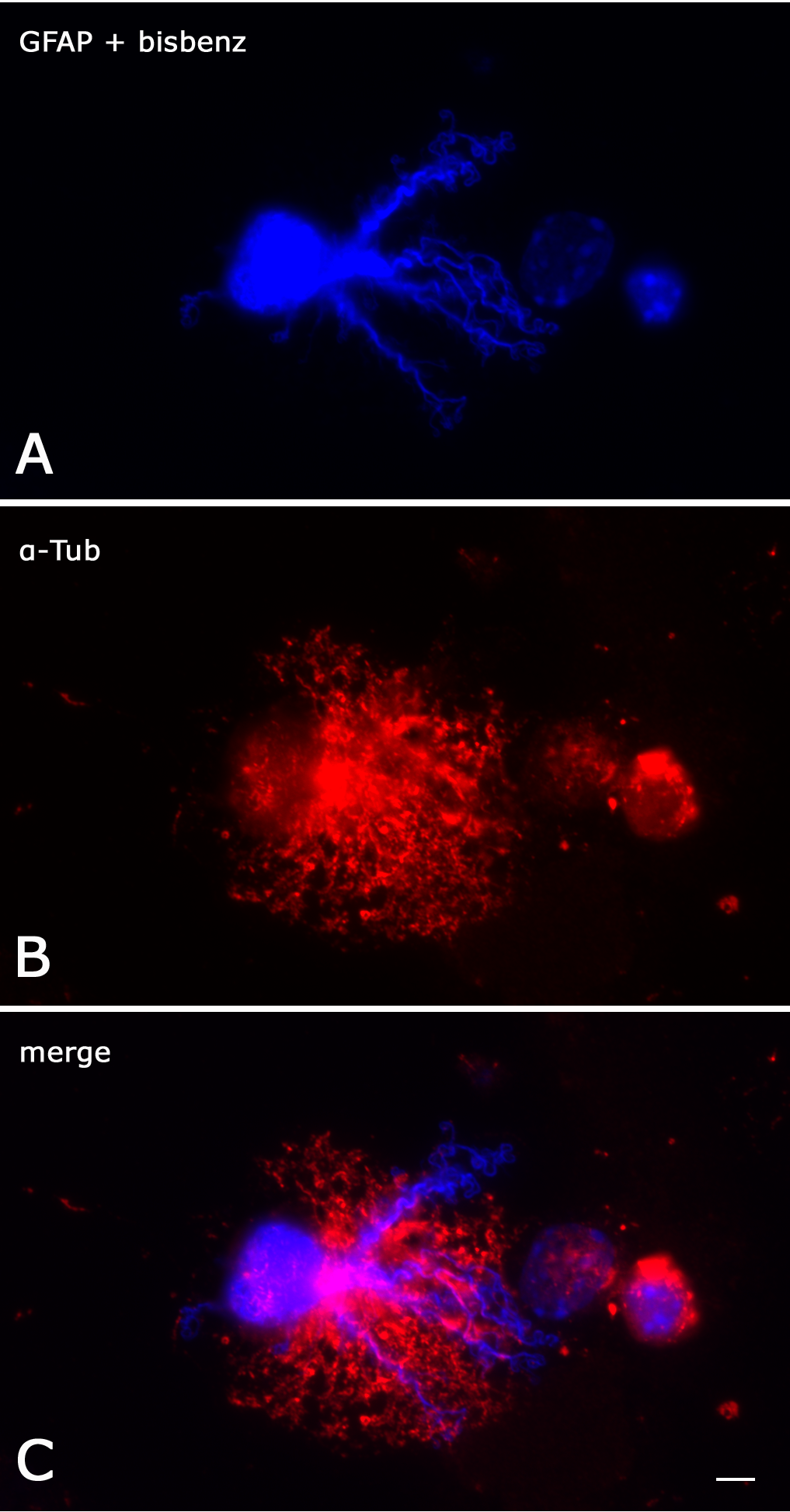

Supplement: Figure S1 — Microtubules are well-preserved in the processes of freshly dissociated, identified astrocytes. Labeling for both, cell nuclei (bisbenzimidine) and glial filaments (GFAP, Alexa 360) is revealed in the blue channel. An astrocyte (center) and two unidentified cells (right) are displayed. Microtubules (α-tubulin, red) are obvious in the astrocyte processes demonstrating that the dissociation method does not interfere with microtubule integrity even in the processes. (TIF) [file pone.0017575.s001.tif]

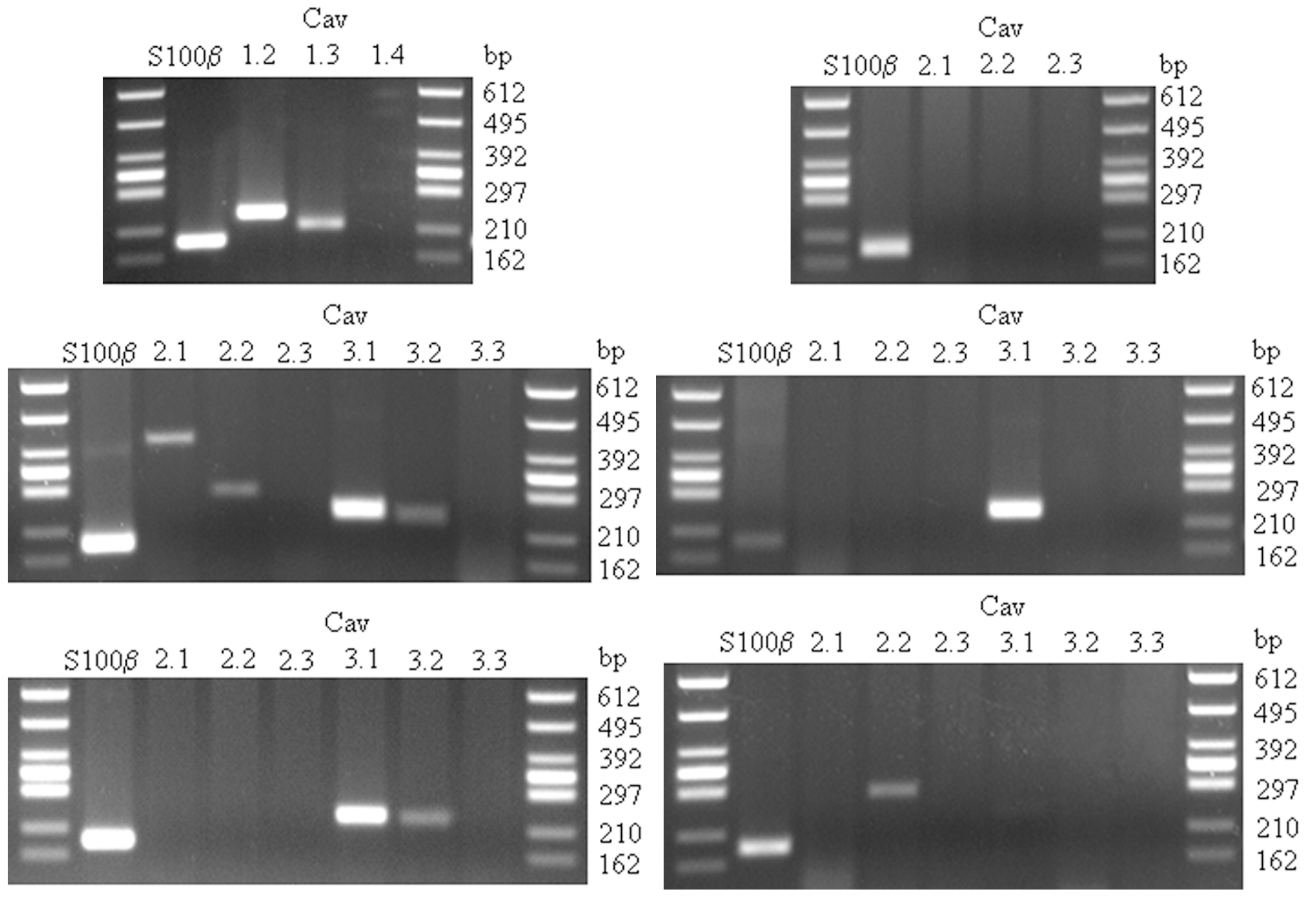

Supplement: Figure S2 — Exemplary agarose gels of mRNA-transcripts for Cav channel family and S100β. (TIF) [file pone.0017575.s002.tif]
