# Supplementary material for: Gray Matter NG2 Cells Display Multiple Ca2+-Signaling Pathways and Highly Motile Processes
Source: PLoS One. 2011 Mar 24;6(3):e17575. doi: 10.1371/journal.pone.0017575 (PMC3063786; doi:10.1371/journal.pone.0017575)
Supplement: Table S1 — Primers used for single-cell RT-PCR. (DOC) [file pone.0017575.s003.doc]

**Table S1: Primers used for single-cell RT-PCR**

| **Gene** | **Primer sequence** | **Product length** | **Position** | **Genbank accession number** |
| --- | --- | --- | --- | --- |
| Cav 1 | se 5‘-GCATTGGGGTCCAGCTBTTCAA as 5’-AAGAAGGCAATGATGATGATRTAGA | 334 bp | 3224, 3158, 3056 3533, 3467, 3365 |  |
| Cav 1.2 (nested) | se 5‘-ATACCTGTTCGGATAGTTCT  as 5’-GATGGGGCCCTTGTCTT | 239 bp | 3257 3479 | NM_009781 |
| Ca_v_1.3 (nested) | se 5‘-TGCAGGGGGCTTTTCATTCTTTAT  as 5’ CACGGTAGTTGTAGACAGGACCAA | 214 bp | 3229 3419 | NM_028981 |
| Ca_v_1.4 (nested) | se 5’-AGGGCTCCTTCCTCATCTACCC as 5’-GATAGGGCCCTCATCTTCTGC | 197 bp | 3131 3307 | NM_019582 |
| Ca_v_2 | se 5’-CCTGGCCACCATCATTGCYAACTG  as 5’-GAGGGGGATGAAGTACARCCAGTT | 718 bp,  715 bp (Cav2.2) | 318, 303, 288 1012, 994, 982 |  |
| Ca_v_2.1 (nested) | se 5’-AGCGGCTGGATGACACGGAACC as 5’-AGCTGGCGACTCACCCTGGATGT | 419 bp | 398 794 | NM_007578 |
| Ca_v_2.2 (nested) | se 5’-CGGGGATTCTCGCCACAGC  as 5’-TTGCCACAGGGAAAGTCACC | 283 bp | 527 790 | NM_001042528 |
| Ca_v_2.3 (nested) | se 5’-CCGTAATGGCTGGAATGTCA as 5’-GATCAAACCCCTCTAGAATACCTG | 323 bp | 471 770 | NM_009782 |
| Ca_v_3 | se 5’-CTTCGTCTTCTTCATCTTYGGCAT as 5’-ATGAAGTAGATGAAGTTGTAGAA | 471 bp (Ca_v_3.1) 486 bp | 657, 714, 645 1105, 1177, 1108 |  |
| Ca_v_3.1 (nested) | se 5’-CTGCAGGAGCGTGCCCACAC  as 5’-ATGATGTCGACCCAGCCCTCCAG | 237 bp | 843 1057 | NM_001112813 |
| Ca_v_3.2 (nested) | se 5’-CCCCAGCCGCCGTGAGCTT  as 5’-CACCCAGCCCTCCAGTGTGATGA | 229 bp | 915 1121 | NM_021415 |
| Ca_v_3.3 (nested) | se 5’-AGGGCCGAGAGTGCTGCCTGT  as 5’-TCTCCACCCAGCCTTCCAGAGTGA | 219 bp | 860 1055 | NM_001044308 |
| vGLUT1/2 | se 5'-CGCTACATCATCGCCATCATGAG as 5'-GGAGGGGCCCATTTGCTCCA | 416 bp | 184, 208 580, 604 |  |
| vGLUT1 (nested) | se 5'-CTGGAGGATTTATCTGCCAAAAAT as 5'-GGTATGTGACCCCCTCCACCAAT | 165 bp | 398  540 | NM_182993 |
| vGLUT2 (nested) | se 5'-ACCGCGGAGGCAAAGTTATCAAG as 5'-AGCAGTATCGCAGCCCCAAAGAC | 169 bp | 314 460 | NM_080853 |
| vGLUT3 | se 5'-TTTGGCTGCTGCAGGCTTATGAGT as 5'-ATGGGAAAATCCAACCACCAGGAG | 443 bp | 830 1249 | NM_182959 |
| vGLUT3 (nested) | se 5'-CCAACTTGGCCAGTCTGAGCA as 5'-ACAGCGGTTGTGGTCAAAATCTTT | 280 bp | 923 1179 |  |
| PDGFα R | se 5'-tcaaagggaggacgttcaagacc as 5'-gacgggcagcacattcatactc | 303 bp | 578 859 | NM_011058 |
| PDGFα R (nested) | se 5'-tgtgtataaggcaggagaaacgat as 5'-tggggacggtcaaagtgta | 167 bp | 669 817 |  |

Position 1 is the first nucleotide of the initiation codon. The length of PCR products was indicated as base pairs (bp). 'Se' und 'as' mark sense und antisense primers. vGLUT 1/2 sense and antisense primers have one mismatch with vGLUT 2, respectively. All sense and antisense primers are located on different exons, respectively.
